# A Multidisciplinary Review of the Microbial, Functional, and Consumer Advancement of Indian *Lassi*

**DOI:** 10.1007/s12602-026-11028-4

**Published:** 2026-05-09

**Authors:** Haran Prasad, Sehyeon Song, Min Ji Jang, Hye Kim, Md Abdur Razzak, Md Ariful Haque, Jaehyun Ahn, Seockmo Ku

**Affiliations:** 1 Department of Food Science and Technology, Texas A&M University, College Station, USA; 2 Department of Agricultural Education and Communication, University of Florida, Gainesville, FL USA; 3 1772 Stadium Rd. Bryant Space Science Center, Room #117B, Gainesville, FL 32611 USA; 4 373 Olsen Blvd. Cater-Mattil Hall, Room #102, College Station, TX 77843 USA

**Keywords:** Indian *Lassi*, Probiotic beverage, Fermentation, Food processing technology, Functional food, Market trends

## Abstract

Indian *Lassi*, a traditional yogurt-based fermented beverage, holds cultural, nutritional, and technological relevance, yet remains comparatively underrepresented in indexed scientific literature. This review synthesizes current evidence on *Lassi’s* composition and fermentation characteristics, emphasizing its probiotic potential and nutrient profile while clearly distinguishing findings derived from *Lassi*-specific studies from those extrapolated from related matrices such as yogurt and buttermilk. Potential health-related effects, including support for gut and immune function and contributions to cardiometabolic risk modulation, are discussed as hypothesized benefits inferred largely from broader fermented dairy research rather than confirmed *Lassi* clinical trials. The diverse variations of *Lassi*, from sweet and salty formulations to spiced desi buttermilk style beverages, are examined alongside regional and global adaptations and their implications for microbial ecology, sensory properties, and consumer acceptance. A bibliometric and text-mining analysis of indexed *Lassi*-related publications maps prevailing research themes, highlighting a concentration on product formulation, quality, and safety, with comparatively sparse human and mechanistic health studies. Emerging technological developments, including precision fermentation, starter culture optimization, and innovative preservation and packaging strategies, are evaluated in relation to microbial stability, regulatory expectations, and cultural authenticity. Collectively, the review identifies critical gaps, particularly the limited experimental validation of *Lassi*-specific functional and health outcomes, and outlines priorities for future work.

## Introduction

### Definition and Historical Context

Indian *Lassi*, also known as desi or traditional Indian buttermilk, is a yogurt-based fermented beverage widely consumed across the Indian subcontinent and other regions, particularly during summer months [1]. Originating in the Punjab region, *Lassi *is available in multiple varieties, including sweet, salted, and mango forms, and is recognized for its probiotic potential [2]. Despite extensive research on fermented dairy products such as yogurt and kefir, scientific literature on *Lassi *remains limited.

*Lassi *has ancient origins linked to early Indian civilizations, with fermented foods documented as early as 4000 BC [3]. Indian dietary practices shaped during the Aryan period viewed food as both functional and health-related, incorporating ritual offerings, seasonal consumption patterns (e.g., *Lassi *in summer), and broad food classifications [4, 5]. The term “*Lassi*” derives from the Indo-Aryan “Lasika” [6], and these traditional practices, including Ayurvedic recognition of yogurt and turmeric for their therapeutic properties, continue to inform health and immune-related applications today [7].

*Lassi *production is an early example of biotechnology, evolving from spontaneous fermentation without starter cultures to controlled processes using selected lactic acid bacteria, molds, and yeasts [8]. Advances in gene technology and synthetic biology now enable these microorganisms to function as cellular factories, while fermented foods overall account for nearly one-third of the global diet [9].

India’s probiotic market has high growth potential, driven by rising health awareness, shifting diets, increasing diabetes rates, lifestyle-related diseases, cardiovascular concerns, and higher incomes [10]. Producing about 132 million tons of milk annually, India transforms 60% into traditional dairy, including *dahi*, *mishti doi*, *shrikhand*, and *Lassi *[11]. These fermented products offer probiotic activity that supports gut, digestive, and cardiovascular health. Enhancing *Lassi ’s *microbial composition, flavor, and appearance could further boost its global appeal.

### Objectives

Indian *Lassi *has long been consumed in India and parts of Europe and is valued for its probiotic potential. Although it has been extensively studied within regional contexts, research on enhancing its probiotic functionality through targeted modulation of microbial composition remains limited, and studies conducted outside South Asia, particularly in the United States, are scarce. Moreover, the long-term health implications of improving probiotic diversity in *Lassi *are poorly understood, partly due to its regional identity and its varied nomenclature, including *chaas*, *mattha*, and Indian buttermilk.

The primary objective of this review is to synthesize existing scientific evidence on the microbial composition, fermentation characteristics, and functional properties of Indian *Lassi*, with an emphasis on identifying knowledge gaps that limit its development as a standardized probiotic beverage. To support this synthesis, a bibliometric analysis of *Lassi*-related literature is presented to map research trends and highlight areas of underrepresentation, drawing where necessary on studies of closely related fermented dairy products such as yogurt and buttermilk. Insights from this analysis are used to inform future directions in fermentation technology and functional enhancement, thereby supporting the evidence-based positioning of *Lassi *within the global probiotic beverage landscape.

## Methods

### Data Collection and Review

We conducted a structured bibliometric search in Web of Science Core Collection and Scopus to identify indexed scholarly publications on Indian *Lassi *and closely related traditional buttermilk beverages (e.g., chaas, mattha). Searches were executed using an equivalent Boolean strategy adapted to each database’s field syntax. In Scopus, we applied the query to TITLE-ABS-KEY; in Web of Science, an equivalent Topic search (title/abstract/keywords) was used.

Search query (Scopus TITLE-ABS-KEY form): (*Lassi *OR chaas OR mattha OR “Indian buttermilk” OR buttermilk) AND (ferment* OR “fermented milk” OR “fermented dairy” OR “fermented milk beverage*” OR “milk beverage*” OR dairy) AND (India OR Indian).

Inclusion approach. Indian *Lassi *is not extensively indexed in global research; we adopted an inclusion-first strategy and avoided overly restrictive exclusions (such as those based on study design, outcomes, or quality score). Records were included in our analysis if they met the following criteria: (i) they were scholarly publications indexed in Web of Science or Scopus, (ii) they focused primarily on *Lassi*, chaas, or mattha (rather than mentioning them incidentally), and (iii) they contained sufficient bibliographic and text metadata for subsequent text mining (including title and abstract, author keywords, and relevant section excerpts where available).

**Fig. 1:**
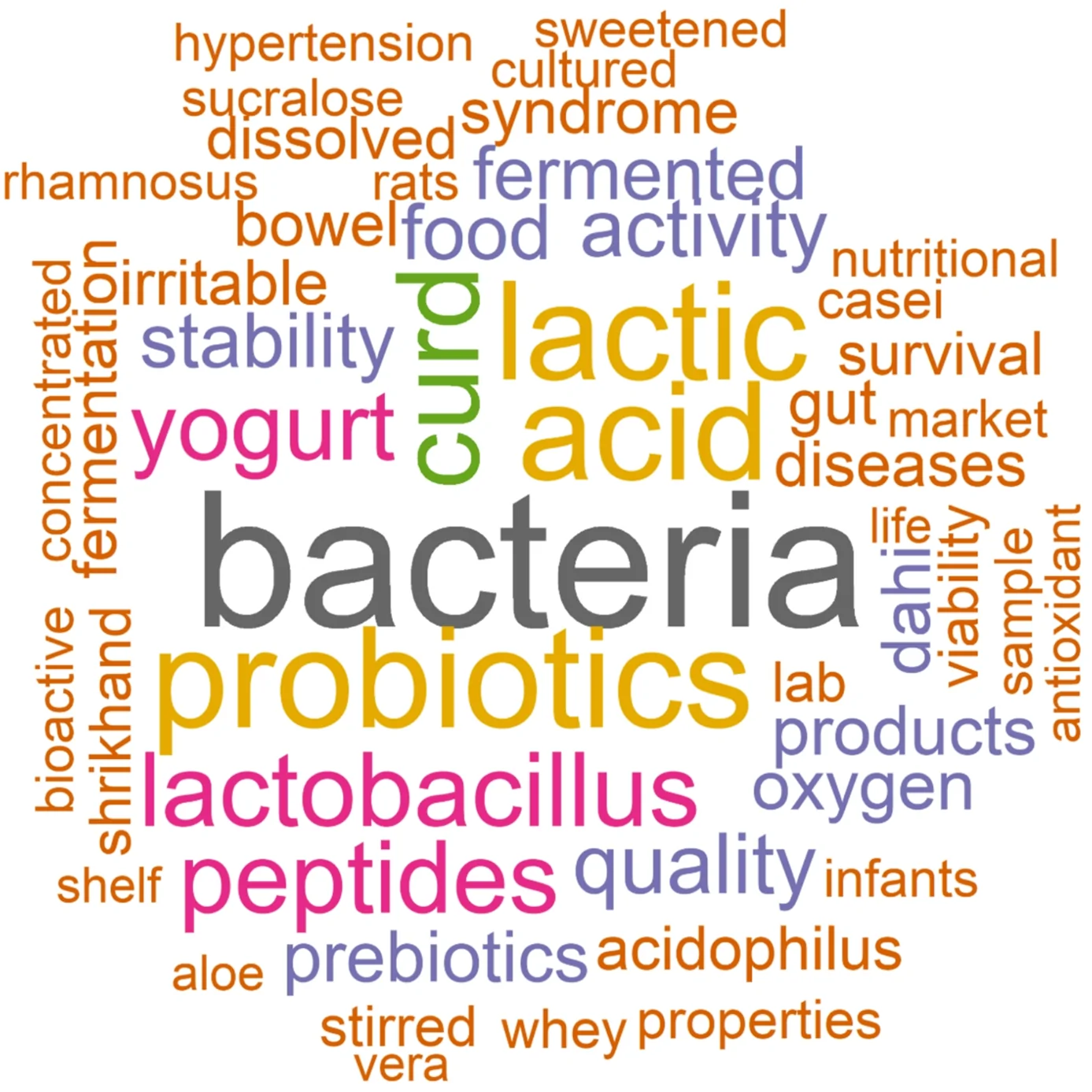
Author keywords were tokenized and aggregated across the analytic corpus. Word size is proportional to the citation-weighted frequency (sum of “Cited” across publications in which the keyword appears), providing a visibility-weighted view of which author-selected concepts are most prominent in the indexed literature. We present this visualization (word cloud), which describes the retrieved corpus, and do not intend it as a definitive statement of field-wide priorities. The intention is to recognize and compare how researchers label their work with how topics emerge from the articles’ prose

**Fig. 2:**
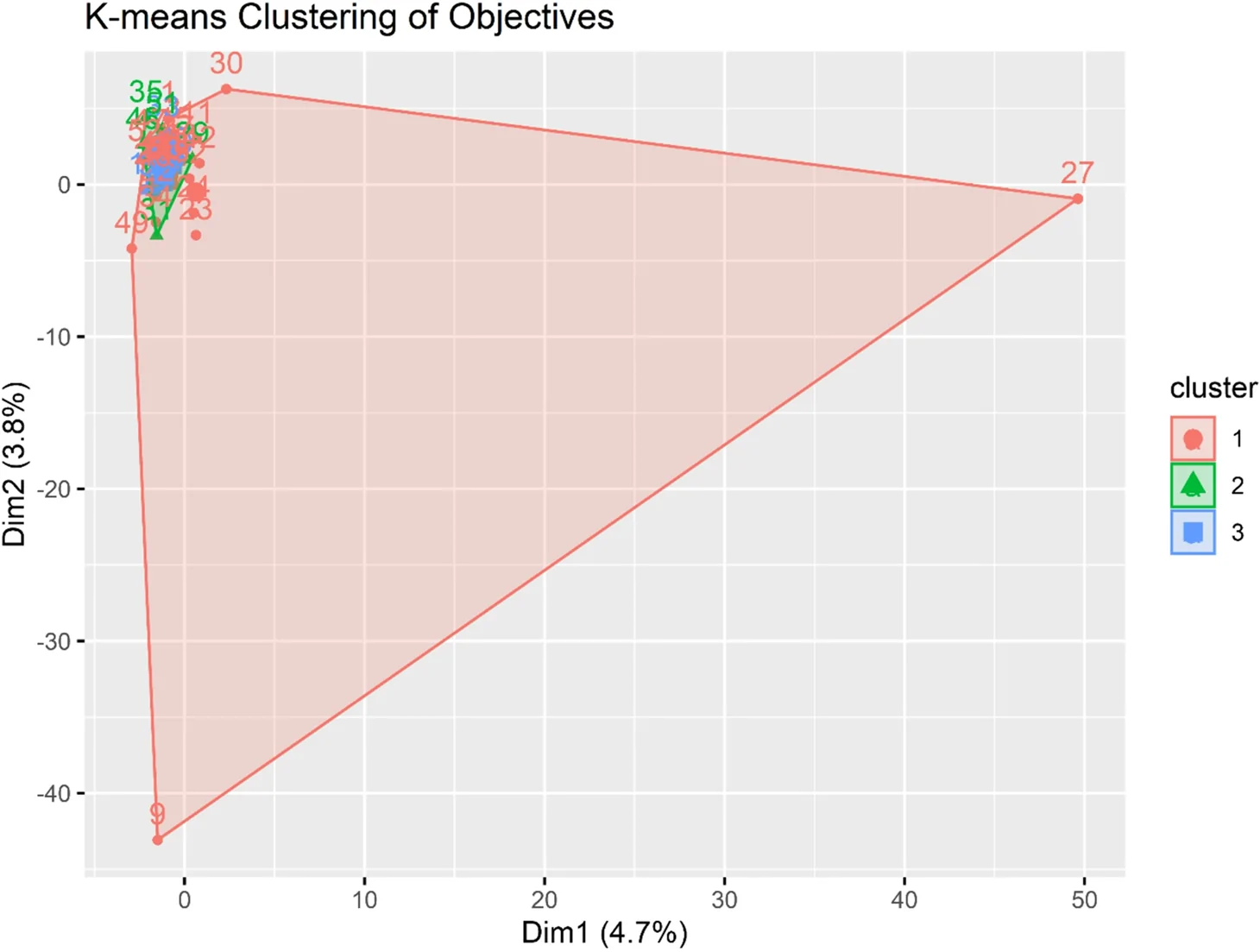
Depicts k-means clustering of the objective’s corpus. We converted each Objective sentence into a document–term matrix and reduced it to a lower-dimensional representation using term frequency–inverse document frequency (TF–IDF). We then applied k-means clustering to group studies with similar stated aims and plotted the resulting clusters, highlighting outlier articles whose objectives differ substantively from the dominant themes. K-means clustering of objectives (k = 3) in the retrieved indexed corpus (*N* = 53). Each point represents one publication, labeled by its dataset record ID, and colors denote cluster membership. The plot shows a two-dimensional projection of a high-dimensional document–term representation; therefore, distances and apparent separation are approximate and are used here to support descriptive summarization rather than to imply sharply bounded subfields. Cluster sizes: Cluster 1 (*n* = 7), Cluster 2 (*n* = 11), and Cluster 3 (*n* = 35). A companion mapping of record IDs to objective text and cluster assignment is provided in Table S2 (or available from the authors upon request if Table S2 is not included)

**Fig. 3:**
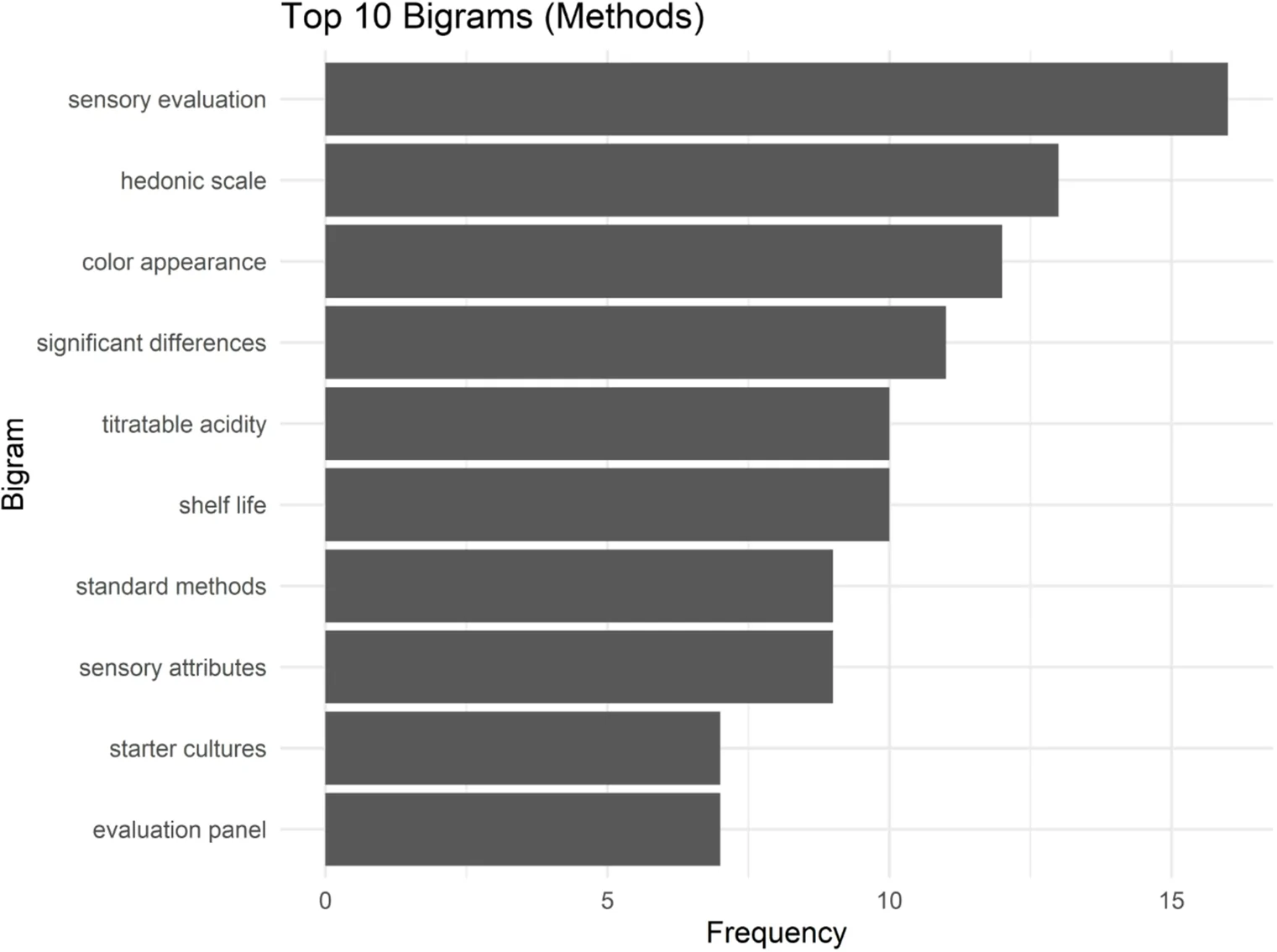
Bigram analysis of the Methods sections. We constructed bigrams (two-word sequences), removed low-frequency and non-informative pairs, and ranked the remaining bigrams by frequency. The resulting bar plot emphasizes recurring methodological phrases. We computed Bigrams from the Methods text after normalization and stop-word filtering. Frequency reflects how often each two-word phrase appears across the corpus and is interpreted as a descriptive indicator of commonly reported methodological elements (e.g., sensory evaluation/hedonic scaling, titratable acidity, shelf-life assessment, and standardized protocols), rather than as a definitive measure of methodological quality or field-wide practice

**Fig. 4:**
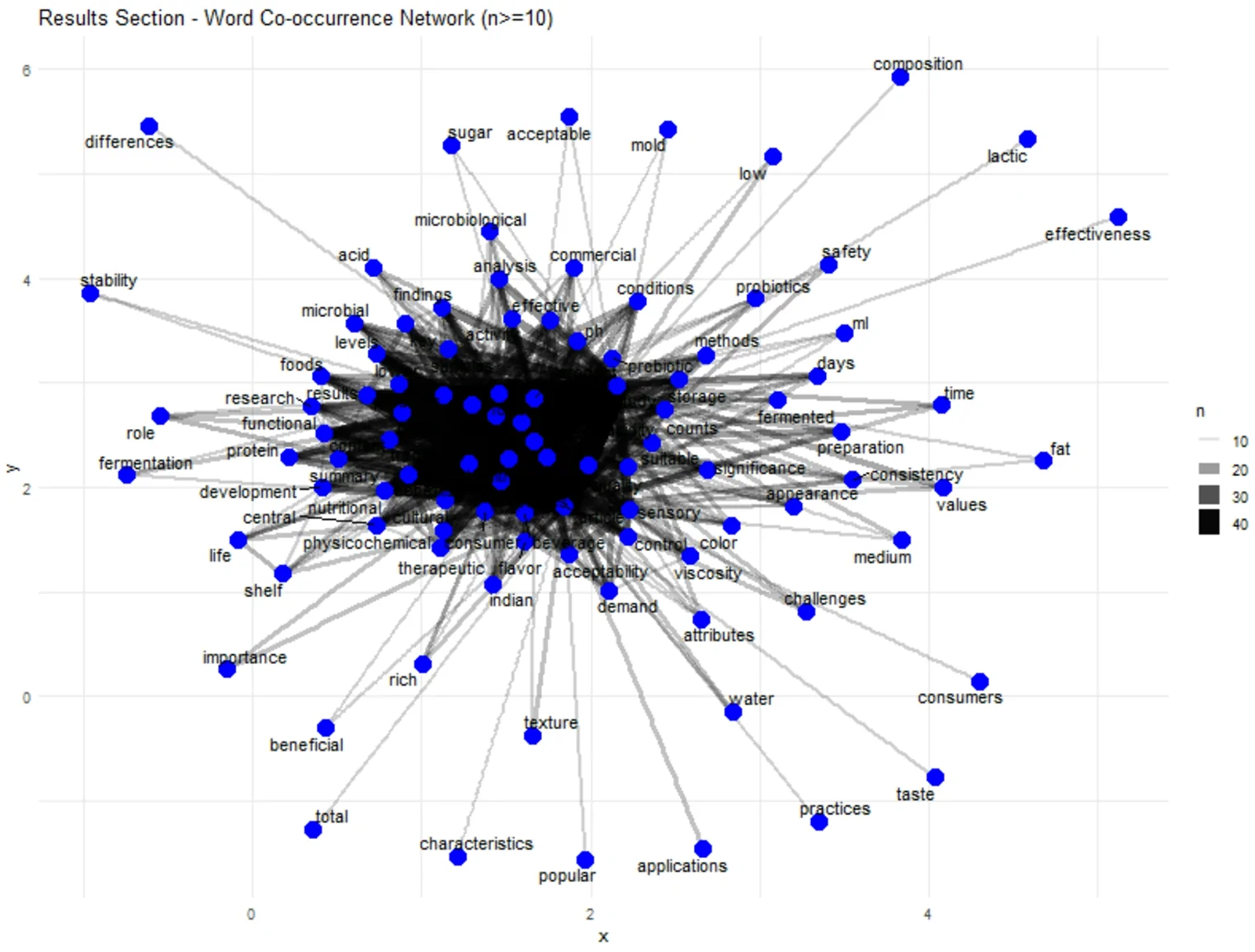
Presents a term co-occurrence network. Results-section word co-occurrence network (*n* ≥ 10) in the retrieved indexed corpus (*N* = 53). Nodes represent terms extracted from the Results text; edges represent term pairs that co-occur within the same publication. Only co-occurrences observed in at least 10 publications are visualized to reduce sparsity and improve readability. The network is undirected and rendered with a force-directed layout; edge width/opacity scales with co-occurrence frequency (n). Distances and apparent clusters are approximate and are interpreted descriptively

Duplicate handling and workflow. Metadata exports from both databases were merged and deduplicated using DOI matching when available, followed by normalized title matching with manual verification of ambiguous matches. Of the 76 identified records (Web of Science = 54; Scopus = 22), six duplicates were removed, resulting in 70 unique records. After applying the minimum eligibility rules above, the final analytic corpus for Figs. 1, 2, 3 and 4 comprised *N* = 53 publications.

Quality considerations. Because the objective is descriptive mapping of themes and publication signals rather than effect-size synthesis, we did not apply a formal risk-of-bias tool. Peer-reviewed indexing and minimum metadata/text completeness were used as quality gates for reproducible text mining.

### Bibliometric Analysis of Indian *Lassi*

Figures 1, 2, 3 and 4 summarize a combined bibliometric and text-mining workflow applied to the final analytic corpus (*N* = 53). For each publication, we compiled a structured extraction table (title, abstract, author keywords, and section excerpts: Introduction, Objectives, Methods, Results, and Conclusions and imported it into R (v. 4.2.3). Text preprocessing included lowercasing, removal of punctuation/numerals, stop-word filtering (including a domain stop list), and part-of-speech filtering using UDPipe to retain content-bearing terms (primarily nouns and adjectives). We extracted key phrases using RAKE, and triangulated results against author keywords to distinguish author framing from salient technical words in the text. Co-occurrence networks were estimated at the document level and visualized as undirected graphs after applying a frequency threshold (e.g., *n* ≥ 10) to improve interpretability.

Our method has limitations. The analytic corpus is modest (*N* = 53), which can make keyword prominence, clustering structure, and co-occurrence topology sensitive to preprocessing decisions (e.g., stop lists) and visualization thresholds. In addition, publication and indexing bias may undercount relevant work published in non-indexed regional outlets, non-English venues, theses, or grey literature. Accordingly, we interpret Figs. 1, 2, 3 and 4 as descriptive signals within the retrieved indexed corpus rather than definitive statements about global *Lassi *research priorities.

Figure 1 (word cloud) summarizes thematic emphasis in the retrieved indexed corpus using citation-weighted author keywords (author framing/visibility). Keywords cluster most strongly around microbiology and functional dairy themes (particularly bacteria/probiotics, lactic acid fermentation, and Lactobacillus), alongside recurring attention to product quality and stability. As a complementary check (not shown), RAKE extraction from abstracts reinforces this emphasis by elevating technically substantive terms such as probiotics and strains, while also surfacing boilerplate phrasing (e.g., present study) that remains non-informative. Altogether, these patterns suggest that, within this modest indexed sample, *Lassi *research predominantly articulates through microbial/functional framing coupled with product-quality endpoints, rather than broader socio-cultural or market systems questions. Because keyword prominence is influenced by citation visibility and preprocessing choices, Fig. 1 serves as a descriptive summary of thematic emphasis in the retrieved corpus rather than a definitive statement of global research priorities. The contrast between author keywords and abstract phrases is therefore best interpreted as a difference in granularity rather than a mismatch between rhetoric and practice.

K-means clustering of the objectives text (k = 3) yields three descriptive thematic groupings within the retrieved indexed corpus (*N* = 53) (Fig. 2). Because the visualization reflects a low-dimensional projection of a high-dimensional document-term matrix, distances and apparent separation should be interpreted as approximate; the clusters are used here to summarize recurring objective types rather than to imply sharply bounded subfields.

The first cluster is dominated by product development and formulation/fortification objectives, where studies aim to develop value-added, synbiotic, or functional *Lassi *by incorporating ingredients such as Aloe vera, honey, tomato pomace, amaranth flour, or curcumin and then optimizing formulations for sensory acceptance, antioxidant capacity, and/or probiotic viability. A second cluster emphasizes probiotic- and health-oriented objectives, including targeted outcomes such as lipid-related markers, obesity-related endpoints in animal models, modulation of enteric infections, or immune-related endpoints via probiotic *Lassi *formulations (e.g. [12, 13]), . The third cluster focuses on quality, safety, and stability objectives, including shelf-life extension, control of spoilage organisms, and surveillance for contamination in market- and street-vended fermented milks, as well as packaging-related safety considerations (e.g., preservative strategies, microbial contamination markers, and material migration concerns) (e.g. [13]), .

Figure 2 also shows a small number of peripheral objectives that are less common in this corpus, such as spray-dried/instant *Lassi *formulations, nanostructured carriers deployed in *Lassi*, carbon nanoparticle synthesis from *Lassi*, or ethnoveterinary uses in livestock contexts (e.g. [14, 15, 16]), . These peripheral cases underscore that the indexed *Lassi *literature spans some highly innovative and context-specific directions; however, given the modest corpus size and indexing constraints, they are best interpreted as emerging niches within the retrieved corpus rather than definitive indicators of field-wide priorities.

High-frequency bigrams (Fig. 3) extracted from the Methods sections emphasize a classical dairy product-testing workflow in the retrieved corpus. The most common pairs are on sensory testing (e.g., sensory evaluation, hedonic scale, sensory attributes, evaluation panel) and standardized quality measurements (e.g., titratable acidity, shelf life, color appearance), with significant differences indicating frequent use of conventional hypothesis testing for formulation or treatment comparisons. Consistent with this orientation, the presence of standard methods suggests a reliance on established dairy/food analytical protocols, and the use of starter cultures reflects recurring attention to fermentation inputs. Advanced analytical terms (e.g., HPLC, mechanistic assays) and designs indicative of large-scale human studies are not prominent among the highest-frequency Method bigrams, suggesting they are less common in this indexed sample rather than absent from literature.

The Results-section co-occurrence network (Fig. 4) provides a descriptive map of which content-bearing terms most frequently appear together within the retrieved indexed corpus. After tokenization and stop-word filtering, we computed document-level pairwise term co-occurrence and visualized an undirected network using an edge threshold (*n* ≥ 10) to improve interpretability. The resulting structure shows a dense central region linking product/quality and sensory-related terms (e.g., quality, sensory/acceptability, flavor/texture, acidity, shelf life/stability) with functional and health-oriented terms (e.g., health, benefits, nutritional, functional, probiotic), suggesting that many studies report outcomes spanning both quality characterization and bio-functional framing. More peripheral nodes reflect less frequently co-occurring, context-specific topics (e.g., processing conditions, market/consumer terms, or specialized measurements). Given the modest corpus size and the dependence of network topology on preprocessing choices and thresholding, Fig. 4 serves as a descriptive signal of thematic co-mention patterns within this indexed sample rather than a definitive representation of field-wide priorities.

In aggregate, Figs. 1, 2, 3 and 4 provide a descriptive summary of thematic emphasis within the retrieved indexed corpus (*N* = 53). The patterns are most consistent with literature centered on product formulation, microbial and functional framing, and quality or safety evaluation, with comparatively fewer signals related to mechanistic health outcomes, gut microbiota or metabolomics, long-term clinical endpoints, or broader dietary and population-health contexts. These observations help explain why many of the health-related discussions in later sections necessarily draw on yogurt and buttermilk studies rather than *Lassi*-specific trials, and they highlight clear gaps that future *Lassi *research should address. In particular, the bibliometric signals point to a need for more longitudinal and human-centered studies, deeper microbiome and metabolite profiling, and stronger linkages between innovative formulation approaches (for example, nanoformulations and instantization) and mainstream quality, safety, and regulatory practices.

## Traditional Formulations and Variations

### Traditional Ingredients

**Fig. 5:**
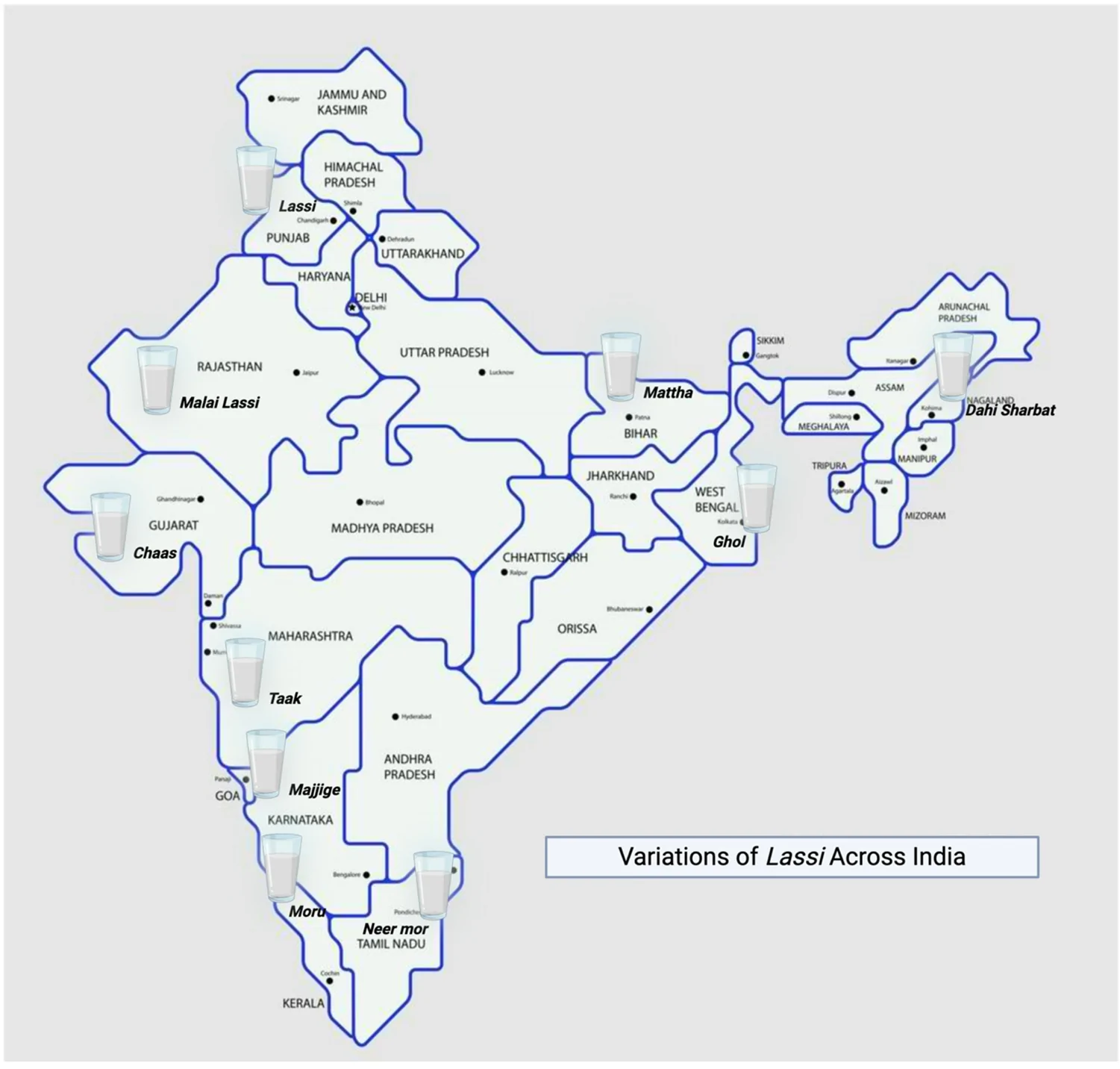
Popular varieties of Indian *Lassi *found across India

The ingredients used in Indian *Lassi *can differ and are based on various factors, such as geographical location, consumer preference, and cultural occasion. Nevertheless, the most basic form of *Lassi *involves two main ingredients: *Dahi* (Yogurt) and water. This creates the foundation upon which additional ingredients can be incorporated into the solution to alter the physio-chemical properties, flavor profile and appearance of the beverage. This has resulted in a variety of *Lassi *products across India and globally. A few commonly consumed *Lassi *varieties are outlined below (Fig. 5). Each individual type of *Lassi *demonstrates a distinct preparation method or ingredient composition which reflects the region’s culture and traditions.

#### Sweet *Lassi*

Sweet *Lassi *contains added sugar, which may come from sucrose, artificial sweeteners, or syrups, giving it a creamy, dessert-like taste. Popular in Northern India, especially Punjab, it is the most widely accepted *Lassi *globally, with variants like Mango and Plain Sweet *Lassi *enjoying high demand in the U.S. market. Its popularity has driven research to enhance its functionality. Moazzem et al. [17] found that sugar level significantly affects *Lassi 's *composition: 15% sugar yielded the highest fat and protein, while 25% sugar had the most total solids and carbohydrates. *Lassi *with 10% sugar had the lowest quality. George et al. [18] reported that sucrose impacts storage, reducing lactic acid while increasing yeast and mold counts, making its stability important for shelf-life improvement.

#### Salty *Lassi*

Salty *Lassi *includes salt and spices, typically cumin, added after fermentation. Popular in Western India, especially Gujarat and Rajasthan, it is less globally accepted than Sweet *Lassi *and is mainly consumed in dry, arid regions during summer. While direct sensory studies on Salty *Lassi *are limited, related evidence from analogous fermented beverages suggests salt-jeera flavored barley fermented drink had the highest acceptability in India, praised for mouthfeel, followed by rose and chocolate flavors [19]. Although conducted on cow’s milk butter rather than *Lassi*, Méndez-Cid et al. [20] demonstrated that salt addition and elevated storage temperatures accelerated lipolytic and oxidative changes, which may have implications for the stability of salted fermented dairy beverages.

#### Spiced Desi Buttermilk (Chaas)

Although spiced desi buttermilk (chaas) differs from *Lassi *in dilution level, fermentation dynamics, and microbial ecology, it is discussed here as a closely related fermented dairy matrix that offers indirect insights into the effects of spices and formulation variables. Skimmed milk powder may be added to increase total solids, improving texture and nutrition. Unlike *Lassi*, which has 10–15% water, buttermilk contains up to 90% water and is popular in Southern India, especially Karnataka and Kerala, where it serves as a cooling drink. Spiced buttermilk is widely accepted in India and is gaining demand in the West, with brands like Amul introducing it in the U.S. market.

Rising demand has prompted studies on spice effects on its physicochemical properties. Sanjay et al. [21] found cinnamon increased carbohydrates, fat, protein, ash, total solids, and acidity, while lowering pH. Cinnamon’s essential oils also showed antifungal and antibacterial activity, extending shelf life. Bule et al. [22] reported Koseret herb reduced oxidation and microbial load while improving sensory acceptance. These findings suggest that specific spices may enhance the physicochemical stability and microbial quality of fermented dairy beverages; however, direct validation in *Lassi *systems remains limited.

### Regional Variants of Indian *Lassi*

Table 1 highlights the diverse regional variations of Indian *Lassi*, showcasing unique flavor profiles, preparation methods, and cultural influences across states. While Punjab’s traditional *Lassi *is rich and creamy, often sweetened or salted, other regions offer spiced buttermilk-style versions like *Chaas* from Gujarat and *Taak* from Maharashtra. Southern states such as Karnataka, Kerala, and Tamil Nadu incorporate curry leaves, ginger, and mustard seeds for distinct aromas and flavors, whereas Bihar and West Bengal balance sweet and savory notes. In the Northeast, *Dahi Sharbat* blends yogurt with honey, lemon, and spices, reflecting local taste preferences. These variations demonstrate *Lassi ’s* versatility as both a refreshing drink and a cultural expression, while also highlighting formulation differences that may influence microbial composition and functional properties.

**Table 1:** Description of the variety of Indian *Lassi *found across different Indian States

Indian State	Name of *Lassi*	Description
Punjab	*Lassi*	Creamy, made sweet with sugar or spiced with salt, cumin, and mint. Served chilled in a Patila glass, a long steel tumbler. Usually topped with cream or butter.
Rajasthan	*Malai Lassi*	Rich, creamy drink, topped with fresh cream (malai) and garnished with nuts, saffron, or cardamom. Often served in kulhads, an earth clay cup.
Gujarat	*Chaas*	Savory spiced desi buttermilk with a high-water content. Seasonings like roasted cumin powder, salt, and fresh herbs such as coriander or mint. Often garnished with green chilies or curry leaves and served chilled.
Maharashtra	*Taak*	Highly diluting yogurt with water and seasoning it with black salt, roasted cumin powder, ghee and sometimes ginger or green chilies.
Karnataka	*Majjige*	Lightly spiced, savory beverage. It is often flavored with ingredients like curry leaves, green chilies, ginger, coriander, and a pinch of asafoetida, giving it a distinct, earthy aroma.
Kerala	*Moru*	Mildly spiced and seasoned beverage. It is infused with flavors of curry leaves, green chilies, ginger, turmeric, crushed garlic, tempering of mustard seeds and dried red chilies in coconut oil. Specially served.
Tamil Nadu	*Neer Mor*	Light and spiced version. Seasoning with ginger, green chilies, curry leaves, asafoetida, and a tempering of mustard seeds. Coriander leaves add freshness, while some variations may include a hint of lime or salt for taste.
Bihar	*Mattha*	Spiced and flavorful beverage. Seasoned with roasted cumin powder, black salt, mint, green chilies, and sometimes ginger. Addition of panchforan (a mix of five spices: fenugreek, mustard, nigella, cumin, and fennel).
West Bengal	*Ghol*	Light and frothy texture. Seasoned with a pinch of black salt and sugar, sometimes a hint of roasted cumin powder for a subtle balance of sweet and savory flavors.
Manipur and Nagaland	*Dahi Sharbat*	Creamy texture with a unique blend of locally available spices and sweeteners. Includes a mix of honey, lemon juice, and a hint of chili or pepper. Some variations contain rose water flavoring.

### Cultural and Religious Significance of Indian *Lassi*

*Lassi *holds a profound cultural and religious significance in South Asia, particularly in India, Bangladesh, and Pakistan, where it features in festivals, rituals, and everyday diets beyond its role as a popular summer beverage. During major celebrations such as Holi, Janmashtami, Diwali, and regional harvest festivals, variations of *Lassi *are commonly served alongside festive foods, symbolizing refreshment, community, and the central place of dairy in regional food culture [23, 24, 25, 26, 27].

Within Ayurvedic tradition, *Lassi *is regarded as a seasonally suitable dietary component associated with digestive comfort and internal cooling, reflecting longstanding health beliefs rather than outcomes established in controlled clinical studies [28]. It is traditionally described as helping to balance the body’s doshas, with sweet *Lassi *considered favorable for Pitta and salted *Lassi *for Vata, and herbs and spices such as cumin, mint, and cardamom are often added to enhance perceived therapeutic value [29, 30, 31].

Overall, *Lassi ’s *embeddedness in festivals, rituals, and traditional medical frameworks illustrates its cultural importance and helps explain its persistence and adaptation as a fermented dairy beverage in both South Asian and global food contexts.

### Global Acceptance of Indian *Lassi*

The Indian diaspora has been central to spreading *Lassi *globally, introducing it through restaurants, food stalls, and grocery stores in regions like North America, Europe, and the Middle East. These communities preserve traditional recipes while promoting varieties such as salted, sweet, and mango *Lassi*. Cultural exchange has increased awareness of *Lassi ’s *traditionally perceived and commercially promoted functional attributes [9], while growing global interest in probiotics and fermented foods has contributed to its popularity as a beverage associated with digestive comfort and cooling effects [32]. As a result, *Lassi *has evolved from a regional specialty to a globally recognized fermented beverage that is often positioned within the functional food and wellness market.

Although Borhani and Moru differ from *Lassi* in formulation, fermentation dynamics, and microbial composition, they are discussed here as culturally and technologically related fermented dairy beverages. In Bangladesh, Borhani, a spiced, savory yogurt drink, is culturally significant at festivals and weddings [33]. In Sri Lanka, Moru, a lightly fermented yogurt drink, traditionally valued for refreshment and digestive comfort [34]. Both share similarities with *Lassi *in ingredients, fermentation, and cultural relevance, reflecting adaptations to local tastes, climates, and resources. Such comparisons highlight how regional factors shape traditional fermented dairy beverages and enhance the study’s originality and scope.

In the U.S. and Europe, Indian culinary influence has led to reinterpretations of traditional foods, including *Lassi*. Mango *Lassi*, blending ripe mangoes with yogurt and sometimes cardamom or saffron, has become especially popular in cafés, smoothie bars, and restaurants. Adapted to local tastes and ingredients, it appeals to consumers seeking novel yet familiar beverages. Its rise also aligns with growing demand for functional, probiotic-rich foods, and in Western markets it is frequently marketed as a healthy, refreshing digestive beverage, reflecting consumer perceptions rather than outcomes established through *Lassi *-specific clinical studies.

Additionally, the use of non-dairy alternatives, such as almond, soy, or coconut milk, in place of traditional yogurt caters to a growing population of lactose-intolerant individuals or those following vegan diets [35]. These Western adaptations not only reflect a growing interest in international cuisines but also demonstrate the versatility of *Lassi *as it is integrated into diverse dietary practices and consumer preferences. As the fusion of traditional Indian ingredients with Western culinary techniques continues, *Lassi ’s *presence in global markets is likely to continue expanding, driven by culinary fusion, consumer interest in fermented foods, and product diversification.

## Functional Profile of Indian *Lassi*

### Phospholipid and Protein Composition

Indian *Lassi *is a fermented dairy beverage that is traditionally considered to have probiotic properties, although direct evidence on *Lassi*-specific functional effects remains limited. These potential health-related effects are hypothesized to be associated in part with its nutrient profile. According to Ali [36], the primary components of *Lassi*, based on dry weight, include protein (31.5–33.1%), lactose (48.7–53.8%), and fat (5.7–13.1%).

*Lassi *contains milk fat globule membrane (MFGM), which is enriched in phospholipids such as phosphatidylcholine, phosphatidylethanolamine, and sphingomyelin. These compounds have been associated with health benefits in other fermented dairy products, but direct evidence for comparable effects in *Lassi *is limited. Based on its MFGM content, *Lassi *is expected to contain higher phospholipid concentrations than milk, and phospholipids make up approximately one-third of the MFGM, contributing to its nutritional profile and hypothesized health-promoting properties [37]. Asif et al. [38] reported that the phospholipid content in buttermilk ranges from 80 to 125 mg/g, which is considerably higher than in whole milk. These measurements were obtained in buttermilk rather than in *Lassi*, and phospholipid levels in *Lassi *have not yet been quantified using comparable methods. A 2019 report by Barukčić et al. [39] likewise showed that buttermilk contains about seven times more phospholipids than whole milk, with concentrations of 0.89 mg/g in buttermilk and 0.12 mg/g in whole milk, and linked this enrichment to desirable emulsifying properties in food applications. Because *Lassi *shares certain technological features with buttermilk, it is plausible that it may exhibit similar phospholipid enrichment and emulsifying behavior, but these assumptions remain speculative in the absence of *Lassi*-specific compositional and functional measurements. Lopez et al. [40] further demonstrated that buttermilk is rich in monounsaturated fatty acids, constituting 48.4% of total fatty acids, with oleic acid representing over 35%, and that it also contains relatively high levels of polyunsaturated fatty acids, including linoleic acid, α-linolenic acid, and docosahexaenoic acid. These findings support a general expectation that fermented dairy systems such as *Lassi *could provide similar lipid profiles, but direct fatty acid analyses of *Lassi *are still lacking and should be prioritized in future work.

Buttermilk, a by-product of butter production, is rich in milk proteins, including both caseins and whey proteins. *Lassi *also contains casein and whey fractions, but systematic comparative studies between buttermilk and *Lassi *are scarce, so direct equivalence in protein composition cannot yet be assumed. The protein profile of buttermilk is comparable to that of skim milk and includes the predominant caseins αs1-casein, αs2-casein, β-casein, and κ-casein, which play a crucial role in determining its structure and texture [41]. Whey proteins, present in smaller quantities in buttermilk, include β-lactoglobulin, α-lactalbumin, immunoglobulins, and serum albumin, and these proteins are well regarded for their nutritional value and functional properties [39]. Buttermilk is also abundant in milk fat globule membrane (MFGM) proteins such as mucin 1, butyrophilin, xanthine oxidase, and adipophilin, which enhance emulsifying properties in buttermilk and have been associated with health benefits in other fermented dairy products; analogous effects for *Lassi *remain to be demonstrated experimentally The concentrations of these proteins in buttermilk vary with milk source and processing conditions, with total protein content generally between 2.4% and 3.5%, and extensive work on buttermilk’s protein composition underscores its technological and potential health relevance, in contrast to the relatively limited protein-focused characterization currently available for *Lassi*.

Buttermilk has a relatively high lactose concentration, reported to range from 3.5% to 4.9% in traditional varieties and from 4.8% to 5.2% in cultured products, whereas galactose levels are much lower than in many other fermented dairy products and average around 0.02%. These values describe buttermilk specifically; comparable lactose and galactose profiles have not yet been characterized systematically for *Lassi*, despite the frequent assumption that they are similar. In buttermilk, the metabolic activity of mesophilic lactic acid bacteria contributes to changes in vitamin levels and can enhance its nutritional profile, but the extent to which analogous vitamin modulation occurs in *Lassi *has not been directly quantified and remains an important topic for future investigation [42].

According to studies by Nirgude et al. [43] and Banerjee and Qamar [44], *Lassi *provides essential nutrients such as calcium, phosphorus, vitamin B2, vitamin B12, pantothenic acid, zinc, potassium, iodine, and molybdenum, which collectively characterize its micronutrient profile. However, these compositional data do not by themselves establish causal links between *Lassi *consumption and specific health outcomes, and such relationships remain largely untested in controlled studies. Furthermore, *Lassi *contains thiamin, niacin, biotin, and folic acid, while being relatively low in iron and vitamin C, which indicates certain nutritional limitations even as it contributes meaningfully to overall nutrient intake within dairy-based diets [42].

### Bioactive Features of Indian *Lassi*

In addition to naturally occurring components such as proteins, lipids, lactose, and micronutrients, *Lassi *may contain other bioactive compounds that arise during fermentation or intestinal digestion, although most evidence for such compounds currently comes from studies on related fermented dairy products rather than from *Lassi *itself. In these other matrices, bioactive constituents can be either naturally present or generated during processing and have been reported to exert physiological and biochemical activities, but direct characterization of comparable molecules in *Lassi *and verification of their in vivo relevance remain very limited. According to González [45], bioactive compounds are substances that, when consumed, can produce physiological and biochemical effects in the human body. Magouz et al. [46] identified 81 peptides in fermented buttermilk, 120 in its gastric digests, and 46 in its intestinal digests, primarily derived from β-casein, αs1-casein, κ-casein, and β-lactoglobulin, including 14 peptides originating from milk fat globule membrane proteins such as lactadherin, butyrophilin, and GlyCAM-1. Among these peptides, 54 have been previously reported to exhibit bioactivity, particularly angiotensin-converting enzyme (ACE) inhibitory activity, yet these findings pertain specifically to buttermilk, and similar peptide profiles in *Lassi *are at present hypothetical, inferred only from shared dairy substrates and fermentation processes. Systematic peptide mapping and functional assays in *Lassi *are therefore needed before such bioactive mechanisms can be confidently attributed to this beverage.

Furthermore, Barukčić et al. [47] reported that whey proteins are rich in essential amino acids and can generate bioactive peptides with potential effects on coronary, gastrointestinal, immune, and nervous system functions, although these findings derive from non-*Lassi *dairy matrices. Such mechanisms may also be relevant for *Lassi *formulations that contain whey-derived fractions, but *Lassi*-specific studies that directly link its peptide profile to defined physiological endpoints have not yet been conducted. In a study by Parekh et al. [48], the bifunctional capabilities of bioactive compounds in fermented whey products were investigated, and cultured buttermilk was found to exhibit several bio-functional properties, including significant angiotensin-converting enzyme (ACE) inhibitory activity, enhanced antioxidant potential, and antimicrobial effects. These observations indicate that fermented dairy systems can serve as carriers of bioactive peptides, but for *Lassi *they should be interpreted primarily as a rationale for future research rather than as evidence that comparable bioactive properties have already been demonstrated.

Taken together, these findings from buttermilk and other fermented dairy systems indicate potential avenues for developing functional fermented food products, including *Lassi*, for introduction into broader markets such as the United States, but they cannot yet be taken as direct evidence of *Lassi *functionality without targeted studies. Incorporation of defined bioactive components into *Lassi *formulations may plausibly enhance its functional properties and could, in theory, influence outcomes such as factors associated with chronic disease, lactose digestion, or mental well-being, yet these possibilities remain hypothetical and will require *Lassi*-specific mechanistic and clinical investigations before any health benefits can be substantiated.

### Short-Chain Fatty Acid Production in Indian *Lassi*

*Bifidobacterium* and *Lactobacillus*, which are commonly used in *Lassi *fermentation, are well documented in other fermented dairy products as contributors to short-chain fatty acid (SCFA) production. In those systems, these bacteria ferment dietary fiber and resistant starch to produce acetic and lactic acids that can serve as substrates for other gut microbes, such as *Faecalibacterium* spp., to generate butyric acid [49]. It is plausible that similar cross-feeding mechanisms operate when *Lassi *is consumed, but direct measurements of SCFA production or flux in response to *Lassi *intake have not yet been reported. Beyond primary fermentation, *Bifidobacterium* and *Lactobacillus* can acidify the gut environment and thereby favor the growth of butyrate-producing bacteria, and propionic and butyric acids are among the SCFAs most frequently linked to gut health and metabolic regulation in broader microbiome research. Lower intestinal pH is also associated with suppression of certain pathogenic taxa and with maintenance of microbial balance, yet for *Lassi *these effects remain hypothetical and are currently inferred solely from analogies to other fermented dairy products rather than from *Lassi*-specific microbiota or metabolite studies.

Short-chain fatty acids (SCFAs) are recognized for important roles in gut and systemic physiology. In various experimental and clinical systems, butyric acid serves as an energy source for colonic epithelial cells and can support gut barrier integrity, while SCFAs more broadly have been implicated in modulation of inflammatory and metabolic pathways. SCFAs may also influence brain function, with studies indicating that butyric acid can affect blood–brain barrier integrity, neurotransmitter levels, and neurotrophic factor expression [50]. These findings, however, derive from general SCFA and fermented food research rather than from *Lassi*-specific studies, so any comparable effects of *Lassi *consumption on gut barrier or brain-related outcomes should currently be viewed as speculative and await direct investigation.

### Microbial Functions and Stability in Indian *Lassi*

Although the microbial dynamics of *Lassi *remain underexplored relative to other fermented dairy products, insights from those systems suggest several plausible functional roles for its microbial communities rather than providing direct evidence. Starter cultures used in fermented dairy products commonly include bacterial genera such as *Lactobacillus*, *Lactococcus*, *Bifidobacterium*, *Pediococcus*, *Leuconostoc*, *Propionibacterium*, and *Streptococcus*, together with yeasts such as *Saccharomyces cerevisiae* and *Debaryomyces hansenii* and filamentous fungi including *Aspergillus*, *Penicillium*, and *Rhizopus* [51]. Yogurt, for example, is typically produced by fermenting milk with Streptococcus thermophilus and *Lactobacillus delbrueckii* subsp. *bulgaricus*, which convert lactose to lactic acid, denature casein micelles, and create the characteristic gel texture [52]. Additional probiotic strains such as *L. acidophilus*, *L. plantarum*, *L. casei*, *L. rhamnosus*, and *L. pentosus* may be incorporated to support putative functional properties, while fruits and flavorings are often added to enhance taste, appearance, and sensory appeal; however, the specific strain combinations, population dynamics, and resulting functions in commercial and traditional *Lassi *products have not yet been characterized with comparable depth.

Given the established microbial effects in related fermented dairy matrices, it is reasonable to hypothesize that similar mechanisms could contribute to the sensory and functional properties of *Lassi*, although this has not yet been demonstrated directly. In yogurt, for example, the fermenting microorganisms determine flavor, texture, color, and aroma, with aroma arising from interactions between milk components and secondary metabolites produced by lactic acid bacteria [53]. Comparable processes may operate in *Lassi *because of the use of related microbial consortia, but strain-specific contributions to *Lassi *sensory attributes have not been systematically quantified. Combinations of lactic acid bacteria shape acidity, taste, and texture, and Ashaolu et al. [54] highlighted the central role of microorganisms in defining fermented foods’ sensory qualities. Similar principles have been observed in other fermented foods; for instance, in North European cured hams, *Tetragenococcus halophilus* and *Staphylococcus equorum* improved color while reducing *Staphylococcus aureus*, and *Staphylococcus xylosus* P2 from Chinese bacon showed promise as a starter culture because of proteolytic, lipolytic, and nitrate-reductive activities that enhanced color, odor, texture, and taste. Collectively, these observations from non-*Lassi *systems suggest that targeted selection of specific strains for use in *Lassi *could, in theory, modulate sensory attributes, functional properties, or safety, but robust experimental evidence confirming such outcomes in *Lassi *products is currently lacking.

Microbial stability during storage is another factor that is likely to be relevant for *Lassi*, as suggested by patterns observed in other fermented dairy products. Dairy products are highly perishable and susceptible to microbial contamination, so appropriate packaging is critical for safety and shelf-life extension, providing protection against temperature fluctuations, light, moisture, gases, odors, microorganisms, pests, and dirt [55]. Historically, plant- and animal-based containers were replaced by wooden boxes, clay pots, and glass, which were later superseded by cardboard cartons and corrugated fiberboards by the early 1900s [56]. Despite such advances, microbial stability can still be compromised during production, transport, or storage because of factors such as temperature, oxygen, humidity, pH, and interactions among microbial cultures. Mani-López et al. [57] reported that during storage, S. thermophilus counts decreased by 1.8–3.5 log units, L. delbrueckii subsp. bulgaricus declined by 30–50%, and probiotic strains such as L. acidophilus, L. casei, and L. reuteri also lost viability. Comparable viability losses are plausible in *Lassi*, particularly when similar starter cultures or probiotics are used, but systematic data on *Lassi *storage stability are sparse, underscoring the need for targeted research on microbial survival and packaging strategies that are specifically optimized for *Lassi *products.

## Health Benefits of Indian *Lassi*

### Probiotic and Thermoregulatory Properties of Indian *Lassi*

**Fig. 6:**
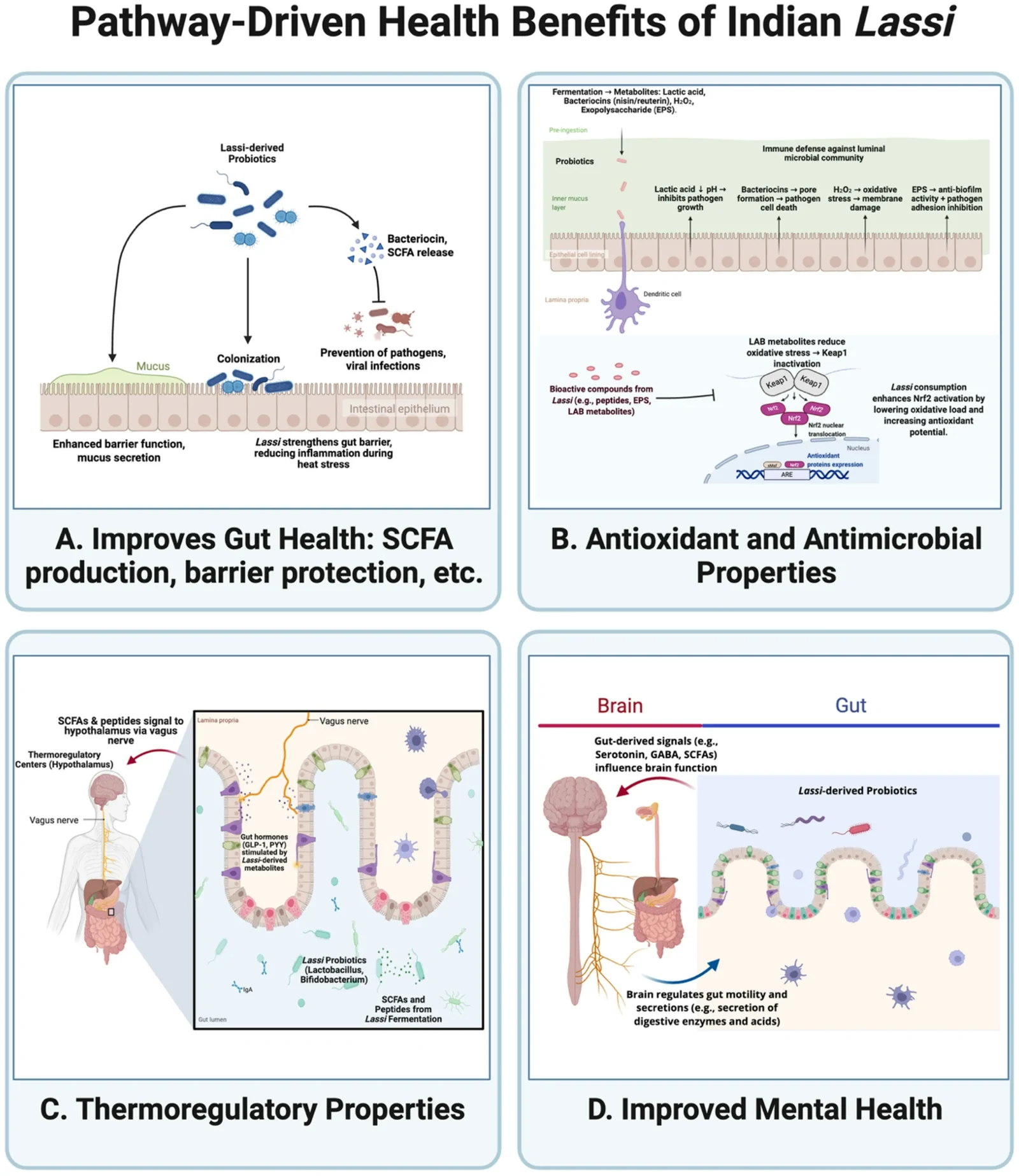
Pathway-driven health benefits of Indian *Lassi *outlining the gut health enhancement, antioxidant and antimicrobial characteristics, thermoregulatory properties and gut-brain axis

As a probiotic beverage, *Lassi *is hypothesized to support various health outcomes (Fig. 6), primarily on the basis of evidence from related fermented dairy products rather than from *Lassi*-specific trials. Proposed effects include antioxidant and antimicrobial activity, modulation of gut health, digestive support, influences on serum cholesterol, and potential impacts on mental well-being and skin health, but these remain putative for *Lassi *because direct clinical studies are scarce [32].

*Lassi *is a rich source of calcium and may contribute to bone health, while offering a dairy option that can be more digestible for some individuals with lactose intolerance [58]. In other fermented dairy products, strains such as *Lactobacillus acidophilus* and *Bifidobacteria* have been reported to alleviate lactose intolerance symptoms by enhancing lactose digestion and modifying gut microbial balance [59]. By analogy, the lactic acid and probiotic content of *Lassi *may support gut microbial balance and digestive comfort, but controlled intervention studies are needed to confirm these effects in *Lassi *consumers. Azarcoya-Barrera et al. [60] reported that lipid-soluble choline from buttermilk enhanced immune system development in rat offspring during lactation and weaning, and Bellés et al. [61] showed that whey- and buttermilk-based formulas helped prevent or restore gut microbiota changes after clindamycin treatment, highlighting their potential as functional food ingredients. Although these findings are not specific to *Lassi*, they point to immune-related bioactivity in similar fermented dairy matrices and provide a mechanistic rationale for future *Lassi*-focused research.

Beyond its proposed probiotic effects, *Lassi *is widely consumed in hot climates for its perceived hydrating and cooling properties (Fig. 6C). Buttermilk, an important component in many *Lassi *formulations, is traditionally regarded as a cooling agent, and this beverage is often used to alleviate sensations of heat and discomfort, including among women experiencing menopausal symptoms such as hot flashes. These cultural practices suggest a thermoregulatory role, but rigorous physiological data on *Lassi *itself are lacking [29]. Lundgren-Kownacki et al. [62] reported that buttermilk consumption was associated with reduced sweat rates and improved subjective perceptions of heat, thirst, and discomfort, indicating potential thermoregulatory and hydration benefits in that matrix. Such findings may be relevant for *Lassi *because of its buttermilk content, yet they should currently be interpreted as indirect support, pending direct measurements of thermoregulation and hydration outcomes following *Lassi *consumption.

### Gut Microbiome Modulation by Indian *Lassi*

While the gut microbiome modulatory effects of fermented dairy products have been extensively studied, research specifically examining *Lassi ’s *impact on gut microbiota remains very limited. Existing studies on fermented dairy products in India often focus on other traditional fermented milk products rather than on *Lassi *itself, so current understanding of *Lassi ’s *microbiome effects is largely inferential. Dedicated *Lassi *intervention studies are therefore needed to elucidate its potential role in gut microbiota modulation.

Gut microbiota dysbiosis has become a concern in the context of modern dietary patterns and pharmaceutical use, motivating interest in strategies for microbiota modulation, including fermented dairy consumption. Fermented dairy products are widely discussed for possible positive effects on gut health, in part because they are often fermented by *Bifidobacterium* and *Lactobacillus*, which can enrich populations of beneficial gut bacteria and may support digestive function in some settings. The magnitude and consistency of these effects, however, vary across studies and are influenced by strain selection, dose, and background diet.

The observed effects of fermented dairy on gut microbiota differ with study design and methodology. Uyeno et al. [63] reported that both probiotic-enriched and non-probiotic yogurt altered gut microbiota composition, although differences between groups were modest and interpretation was constrained by the lack of sequencing-based profiling. In elderly Japanese adults, regular intake of yogurt containing *Lacticaseibacillus paracasei* Shirota (three or more times per week) improved microbiota stability, whereas lower intake was associated with greater temporal fluctuations [64]. Volokh et al. [65] found that 30 days of yogurt fortified with *Bifidobacterium animalis* subsp. *lactis* increased *Bifidobacterium*, *Adlercreutzia equolifaciens*, and *Slackia isoflavoniconvertens*, enhanced markers of lactose metabolism and amino acid synthesis, and reduced predicted lipopolysaccharide biosynthesis, suggesting possible anti-inflammatory potential. These studies clarify how specific fermented dairy matrices and strains can shape the microbiome, but they do not yet establish functional equivalence for *Lassi*; analogous trials using *Lassi *formulations would be required to do so.

Importantly, not all fermented dairy products induce measurable microbiota shifts, and observed effects depend strongly on experimental conditions and probiotic delivery vehicles. For example, one study comparing delivery of *Bifidobacterium animalis* subsp. *lactis* via yogurt or capsules found that yogurt increased the relative abundance of *Bifidobacterium* more than capsules, yet overall gut microbiota composition and fecal short-chain fatty acid concentrations changed little, underscoring the modest impact of some interventions and the importance of the dairy matrix for probiotic survival [66].

A study in postmenopausal Latvian women reported that daily yogurt consumption (175 g for eight weeks) did not produce major microbiota differences between yogurt and control groups, whereas habitual dietary patterns showed clearer associations with microbial diversity. Higher intake of grains and grain-based products correlated with increased *Bifidobacterium* abundance, while greater consumption of meat and processed foods was linked to shifts in the Firmicutes-to-Bacteroidota ratio, suggesting that background diet can have a stronger influence on gut microbiota than yogurt intake alone [67].

Collectively, these findings indicate that fermented dairy products can modulate gut health, but their effects are context dependent and shaped by interactions among probiotic strains, host diet, and the structural and nutritional properties of the dairy matrix. The presence of substrates that support production of health-relevant metabolites and enhance probiotic persistence appears critical for meaningful microbiota modulation. Future studies should therefore evaluate how dietary patterns interact with probiotic-containing fermented dairy products to influence gut microbiota profiles. *Lassi*, a fermented dairy beverage that often contains *Bifidobacterium* and *Lactobacillus*, might be expected to exert similar gut modulatory effects, yet this expectation currently rests almost entirely on extrapolation from other products. Focused research on *Lassi ’s *impact on gut microbiota, including assessments of probiotic survival, colonization potential, and metabolic outputs relative to other fermented dairy products, is needed to determine whether *Lassi *can induce comparable microbiota changes and to define its role in strategies for gut health improvement.

### Microbiome-modulating Effects of Spices in Indian *Lassi*

**Fig. 7:**
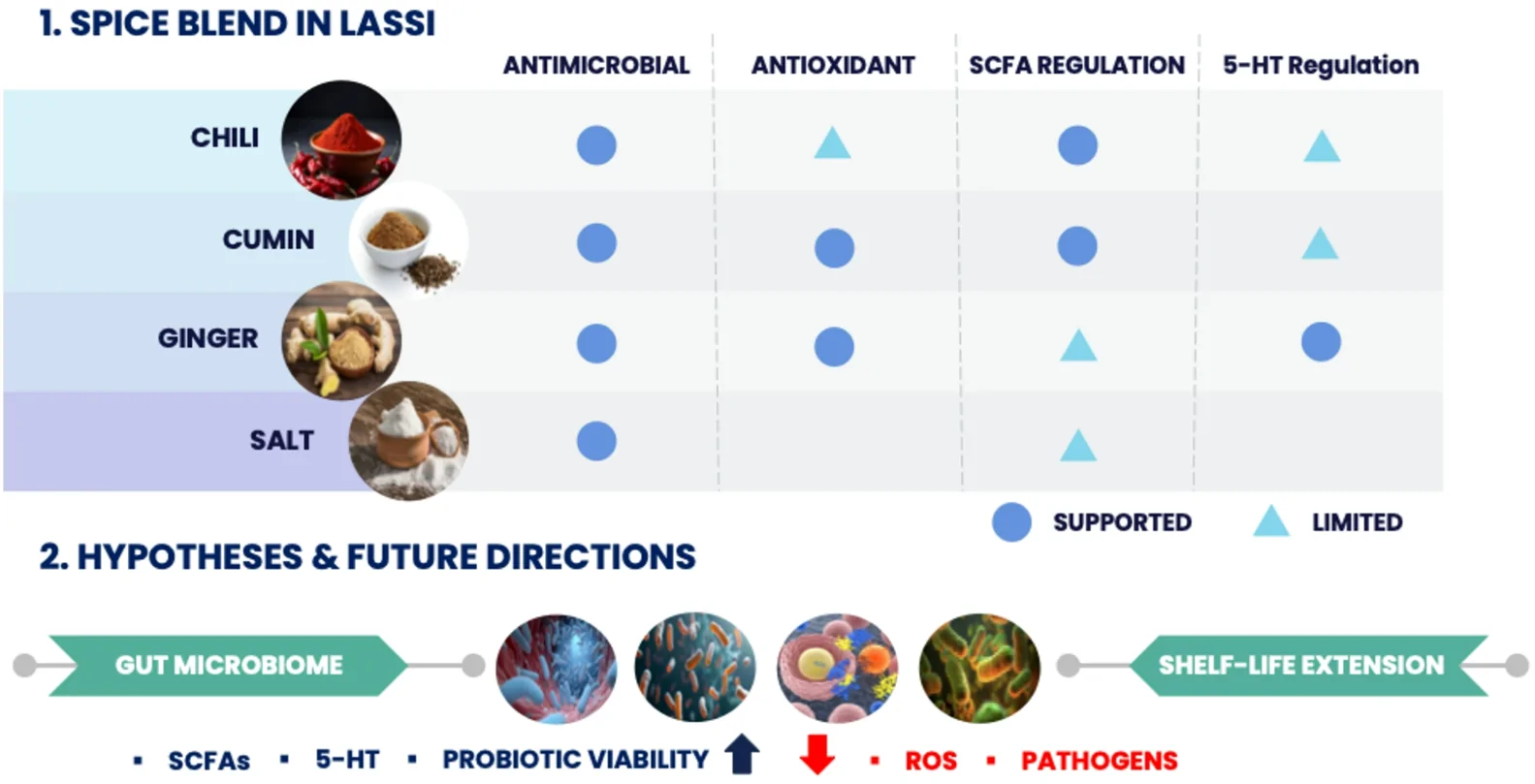
Overview of Common *Lassi *Spices: Functions and Future Insights

Although regional variations exist, *Lassi *has traditionally been supplemented with a variety of spices and functional ingredients. Among these, chili, cumin, ginger, and salt are commonly used across many regions (Table 1). These additions are intended not only to enhance flavor but also to contribute to perceived health benefits and improved shelf life, although systematic scientific validation of these functional roles in *Lassi *remains limited, particularly with respect to microbial stability and product preservation. Clarifying how different spices influence microbial community structures in both the gastrointestinal environment and the *Lassi *matrix, and elucidating their mechanisms of action in modifying product stability, therefore represents an important direction for future research (Fig. 7).

Experimental studies indicate that chili pepper can affect gut microbiota composition, SCFA profiles, and serotonin (5-HT) levels, with potential implications for gut-brain interactions, but these observations come from general food research and have not been confirmed in *Lassi *formulations [68]. Chili pepper has also demonstrated antioxidant and antimicrobial activities, supporting its exploration as a functional food ingredient [69]. Cumin has been reported to regulate SCFA composition in human fecal samples, alter gut microbial distributions, and enhance antioxidant capacity [70], and some studies describe antibacterial properties as well [71]. Spices from the Zingiberaceae family, including cardamom, turmeric, and ginger, show various antioxidant effects [72] and antimicrobial activities, and ginger in particular has been associated with sex-specific changes in gut microbiota composition [73]. These findings collectively suggest that chili pepper, cumin, and ginger can provide antioxidant activity largely through their polyphenol content, and that salt continues to play a classical preservative role in foods such as cheese and reformulated meat products. At the same time, high-salt diets have been linked to hypertension and to salt-associated alterations in gut microbial composition, indicating potential trade-offs that must be considered when formulating salted *Lassi *products [74].

Taken together, the health-promoting and preservation-enhancing properties reported for these spices in other food systems may contribute to the overall quality of *Lassi*, but this contribution has yet to be quantified in a rigorous, *Lassi*-specific context. Polyphenol-based antioxidant activity could, in principle, prolong the shelf life of fermented dairy products by limiting oxidative degradation and supporting microbial stability under storage conditions, and antimicrobial effects may help reduce contamination by spoilage organisms, thereby improving hygienic quality and safety. Future studies should systematically evaluate how these spices influence both the microbial ecosystem of *Lassi *and its shelf-life characteristics, using designs that can disentangle spice effects from those of the underlying dairy matrix. An integrative research approach that combines microbiological, biochemical, and functional assessments will be essential to determine whether spiced *Lassi *can be positioned as a modern functional food on the basis of robust experimental evidence rather than inferred benefits.

### Clinical Research

Clinical research specifically on Indian *Lassi *is very limited, which constrains the strength of inferences that can be drawn in this review. Most mechanistic and clinical insights discussed here are inferred from studies on other fermented dairy products, particularly buttermilk, and should not be interpreted as direct evidence for *Lassi *without validation. Targeted human investigations are required to clarify *Lassi ’s *bio-functional potential.

#### In-vitro Research

Dhameliya et al. [75] investigated the in vitro cholesterol-lowering potential of probiotic bacteria isolated from buttermilk, screening isolates for bile salt deconjugation and cholesterol removal using plate assays, thin-layer chromatography, and cholic acid estimation. The highest cholesterol removal occurred in the presence of 0.3% bile, with co-precipitation of cholesterol and cholic acid, and three potent isolates identified by 16 S rRNA gene sequencing were *Lacticaseibacillus paracasei* (PGB01), *Lactiplantibacillus plantarum* (PGB02), and *L. paracasei* subsp. *tolerans* (PGB05). These findings highlight the in vitro potential of buttermilk-derived probiotics for cholesterol reduction and provide strain-level leads that could be explored in *Lassi *matrices, although such experiments have not yet been reported. *Pediococcus pentosaceus* OBK05 from buttermilk also showed strong α-amylase (72%) and α-glucosidase (61%) inhibition, lowered cholesterol levels, and inhibited HT-29 colon cancer cell proliferation by 87.57% through induction of G1-S phase arrest, further illustrating the range of bioactivities that may be achievable in fermented dairy systems but remain to be tested directly in *Lassi* [76].

#### In-vivo Research

Bellés et al. [77] found that a buttermilk-based formula enriched with lactoferrin and milk fat globule membrane (MFGM) components reversed clindamycin-induced dysbiosis in mice, restoring gut motility, normalizing TLR2 and TLR4 expression, and reducing oxidative stress, which suggests that buttermilk-enriched functional foods can aid gut and immune recovery after antibiotic disruption. These experiments provide relevant context for the potential of *Lassi*-like matrices but do not include *Lassi *itself, so any application to *Lassi *remains speculative. Azarcoya-Barrera et al. [60] reported that buttermilk-rich, phosphatidylcholine-heavy diets enhanced T cell function, promoted a Th1-dominant immune response, increased IL-10 production, and supported postnatal growth, underscoring the value of buttermilk in maternal and infant nutrition. Bhukya and Bhukya [76] showed that *Pediococcus pentosaceus* OBK05 reduced cholesterol, decreased lipid accumulation, improved intestinal histology, and slowed tumor progression in mice, indicating potential for managing diabetes, hypercholesterolemia, and cancer, although translation to humans will require clinical trials. Together, these in vivo data frame a plausible functional space for *Lassi *formulations but stop short of demonstrating *Lassi*-specific effects.

#### Clinical Studies

Mane et al. [78] examined the effects of rectal administration of medicinal plant-processed buttermilk on gut microbiota and weight in obese individuals, observing transient reductions in body measurements and blood glucose along with microbiota changes that largely reverted by day 45, which suggests that repeated or prolonged administration might be necessary for sustained benefits. Conway et al. [79, 80] conducted double-blinded, randomized, placebo-controlled crossover trials to evaluate buttermilk consumption in adults with moderate LDL cholesterol, and found reductions in total cholesterol, LDL cholesterol, and triacylglycerols, alongside increased plasma lathosterol concentrations indicative of enhanced cholesterol synthesis and β-sitosterol-associated inhibition of intestinal cholesterol absorption. Buttermilk intake also led to significant reductions in systolic and mean arterial blood pressure and decreased plasma angiotensin I-converting enzyme levels, without changes in angiotensin II or aldosterone, supporting a role for buttermilk as a cardiometabolically relevant functional food.

At present, clinical evidence on *Lassi *itself is essentially absent. Human studies on buttermilk demonstrate effects on cholesterol, blood pressure, and gut microbiota [78–80], but these outcomes cannot be assumed to apply to *Lassi *without dedicated trials that account for its distinct formulations and consumption patterns. Given that *Lassi *contains probiotics, bioactive peptides, and functional nutrients, the most urgently needed investigations include randomized controlled trials, dose-response interventions, and long-term follow-up studies to evaluate gut microbiota modulation, metabolic and cardiovascular outcomes, and immune responses in populations that consume *Lassi *regularly.

### Extended Application in Neurobiology

Some studies have suggested that fermented food products, including beverages similar to *Lassi*, may influence longevity and aspects of the microbiota-gut-brain axis [81], although direct evidence linking *Lassi *consumption to such outcomes is currently lacking. Fermented foods such as *Lassi *are characterized by rich microbial communities and metabolites, which can include neurotransmitters, neuroactive compounds, and neuromodulators that are theoretically capable of engaging immune, neuroendocrine, enteric nervous, and circulatory pathways within the microbiota-gut-brain axis, thereby influencing brain function and well-being. A study by Buey et al. [82] highlighted the potential of dairy by-products, including whey and buttermilk, to modulate central nervous system inflammation and promote brain health by altering serotonin activity and immune responses, with possible implications for neuroinflammation, mood disorders, cognitive challenges, and neurodegenerative diseases. Similarly, a study by Davies et al. [83] reported that milk fat globule membrane (MFGM) supplementation affected stress and psychological outcomes, with reductions in anxiety and improvements in general psychological health that extended beyond acute situational stress. These observations collectively support a plausible neurobiological role for components that are also present in *Lassi*, but until *Lassi*-specific mechanistic and clinical studies are undertaken, any claims about its effects on longevity or microbiota-gut-brain axis function should be regarded as speculative.

## Comparative *Lassi *Processing Methods

Lassi is produced through two primary approaches, traditional household preparation and modern industrial processing, each characterized by distinct practices and levels of standardization. Traditional methods typically involve natural fermentation of milk using locally maintained starter cultures, followed by manual churning, which often leads to variability in texture, flavor, and microbial composition. In contrast, industrial production relies on standardized operations, including controlled pasteurization, homogenization, and the use of defined starter cultures, to achieve consistent quality, safety, and large-scale output. These two preparation pathways illustrate the technological progression of *Lassi *production while maintaining its cultural relevance as a fermented dairy beverage.

### Traditional Preparation Methods

The traditional method of *Lassi *production varies across households, with each having its own distinct recipe. Typically, traditional *Lassi *is made by whisking *dahi* (plain yogurt) until a smooth texture is achieved. Cold water is then added and mixed until the yogurt and water form a homogeneous solution. The *dahi* is churned using a hand-held instrument called a *madhani*, which serves to separate milk fat and prepare butter. An earthen pot is often used to both prepare and store the *Lassi *[84]. In India, *dahi* is traditionally made using mesophilic starter cultures, including *Lactococcus lactis* subsp. *lactis* and *Lactococcus lactis* subsp. *cremoris*, in a 1:1:1 ratio, along with flavor-producing *Leuconostoc* spp., and incubated for 18–24 h at 23–24 °C. In contrast, *dahi* fermentation typically occurs more slowly than yogurt fermentation, with acid production being slower compared to yogurt, which usually ferments within about 5 h [85]. Depending on individual preferences, additional ingredients such as cumin powder, salt, or sugar can be added to *Lassi *to enhance its flavor.

### Modern Industrial Processing Methods

**Fig. 8:**
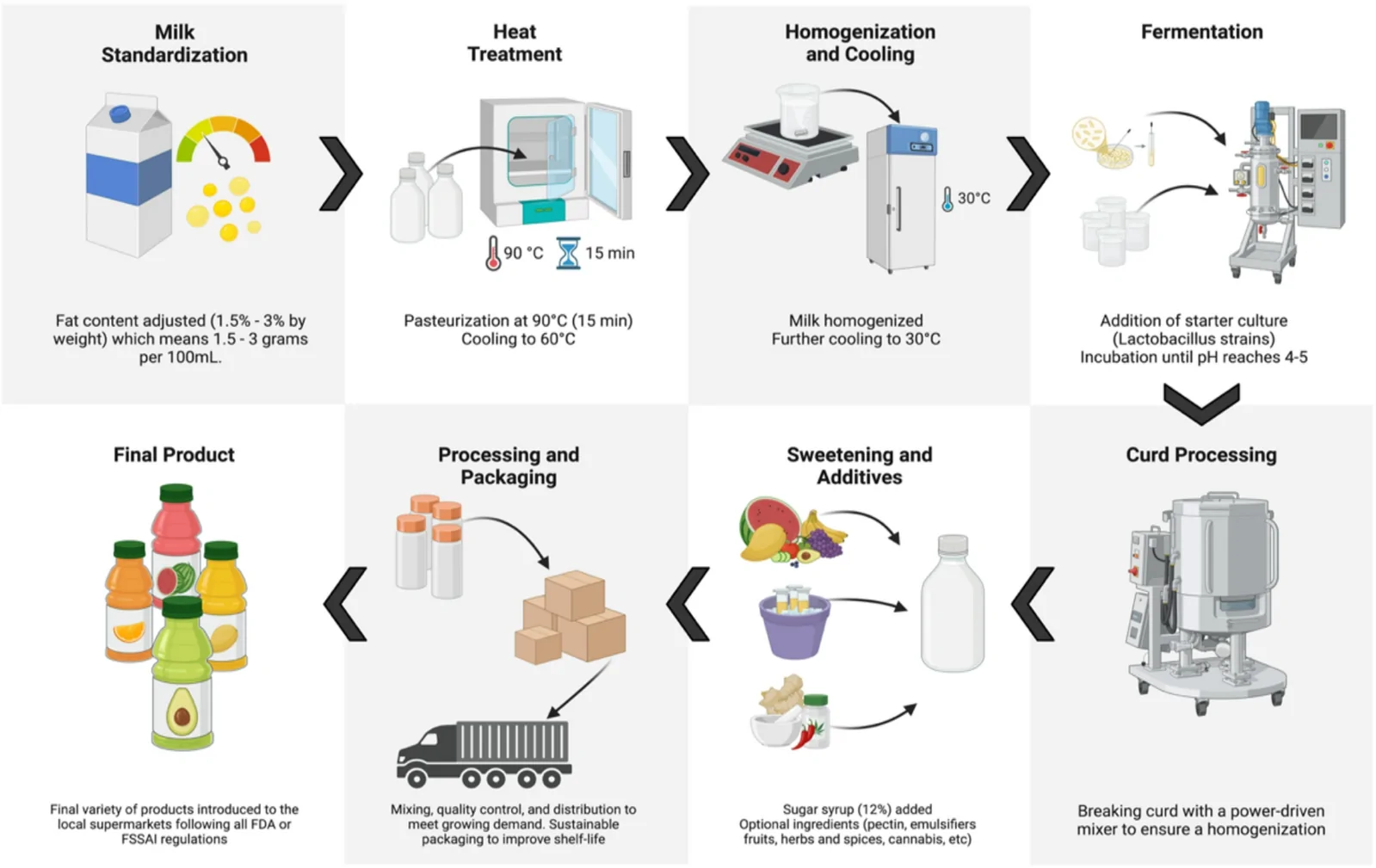
Flowchart of the industrial *Lassi *production process, detailing key steps from milk standardization to final packaging

On the contrary, the industrial *Lassi *production method is complex and expensive due to the requirement of supplying significantly larger volumes of *Lassi *for distribution purposes in the current market (Fig. 8).

As previously mentioned, the rate of acid production in *Dahi* is relatively slower than yogurt fermentation. Therefore, commercial processors are increasingly utilizing yogurt cultures, specifically *Streptococcus thermophilus* and *Lactobacillus delbrueckii* subsp. *bulgaricus*, to prepare *dahi* [85]. This shift is driven by the faster acid production and consistent fermentation results that these yogurt cultures offer, making them more suitable for large-scale commercial production. Standardized milk, with a typical fat range between 1.5% and 3%, is heated and pasteurized to 90 degrees Celsius for 15 min and later cooled down to 60 degrees Celsius. Subsequently, the pasteurized milk is homogenized and cooled further down to 30 degrees Celsius. Once cooled, the starter culture, mostly consisting of *Lactobacillus* strains, is introduced into the milk and allowed to incubate until a slightly acidic pH, of approximately 4 to 5, is achieved. Post fermentation, the curd is broken with the help of a power-driven mixer and sugar is added at a 12% sugar syrup concentration. Based on the company and product development preferences, additional ingredients can be introduced to the *Lassi*, such as low methoxy pectin to improve appearance and texture, artificial fruit-flavoring, herbal spices, etc. [86].

## Market Validity of Indian *Lassi*

### Current Market Trends for Indian *Lassi*

The *Lassi *market is expanding globally, fueled by shifting consumer preferences and interest in functional foods. South Asia accounts for about 65% of consumption, with India’s RTD dairy beverage market, valued at INR 56 billion in 2024, projected to grow at a 16.96% CAGR to INR 143.45 billion by 2030 [87]. Growth is strongest in urban and semi-urban areas. Internationally, demand for functional dairy drinks is rising, with the global market valued at USD 243.22 billion in 2024 and expected to reach USD 463.19 billion by 2033 [88]. Ayurveda-inspired varieties, such as turmeric- and cardamom-infused *Lassi*, are boosting exports, which have grown over 19% annually since 2020 [89].

The RTD market has emerged as a dominant driver of growth in the *Lassi *market. Although no sources indicate the global RTD *Lassi *market value, recent market reports suggest the global buttermilk powder market was valued at approximately USD 3.25 billion in 2024 and is projected to grow at a CAGR of 6.2% to 2030 to reach USD 4.66 billion [90]. This growth is a result of the increasing demand for convenient and nutritious beverage options, particularly in urban areas. Leading brands, such as Nestlé, Danone, and Unilever, have diversified their offerings by introducing innovative packaging formats, such as shelf-stable tetra packs and eco-friendly PET bottles, which have significantly enhanced distribution capabilities and product longevity [91]. Furthermore, the availability of unique flavors, such as mango *Lassi*, masala buttermilk, and fusion varieties incorporating ingredients like chia seeds and turmeric, has expanded consumer appeal.

Health-conscious trends are driving innovation in organic, low-fat, and sugar-free *Lassi*. About 60% of consumers prefer organic dairy beverages for their nutritional quality, lower chemical exposure, and sustainability [92]. Demand for low-fat and sugar-free options is rising alongside global increases in obesity and diabetes, with over 530 million adults living with diabetes [93]. Brands are responding with natural sweeteners like stevia and low-calorie variants for diet-conscious consumers.

The global dairy beverage market size reached a valuation of USD 81.48 billion in 2024, and the market is projected to exhibit growth at a CAGR of 5.10% up to 2030 to reach USD 109.82 billion [94]. These functional products are marketed as aiding gut health, immunity, and overall wellness, resonating with the growing consumer preference for preventive healthcare solutions.

**Fig. 9:**
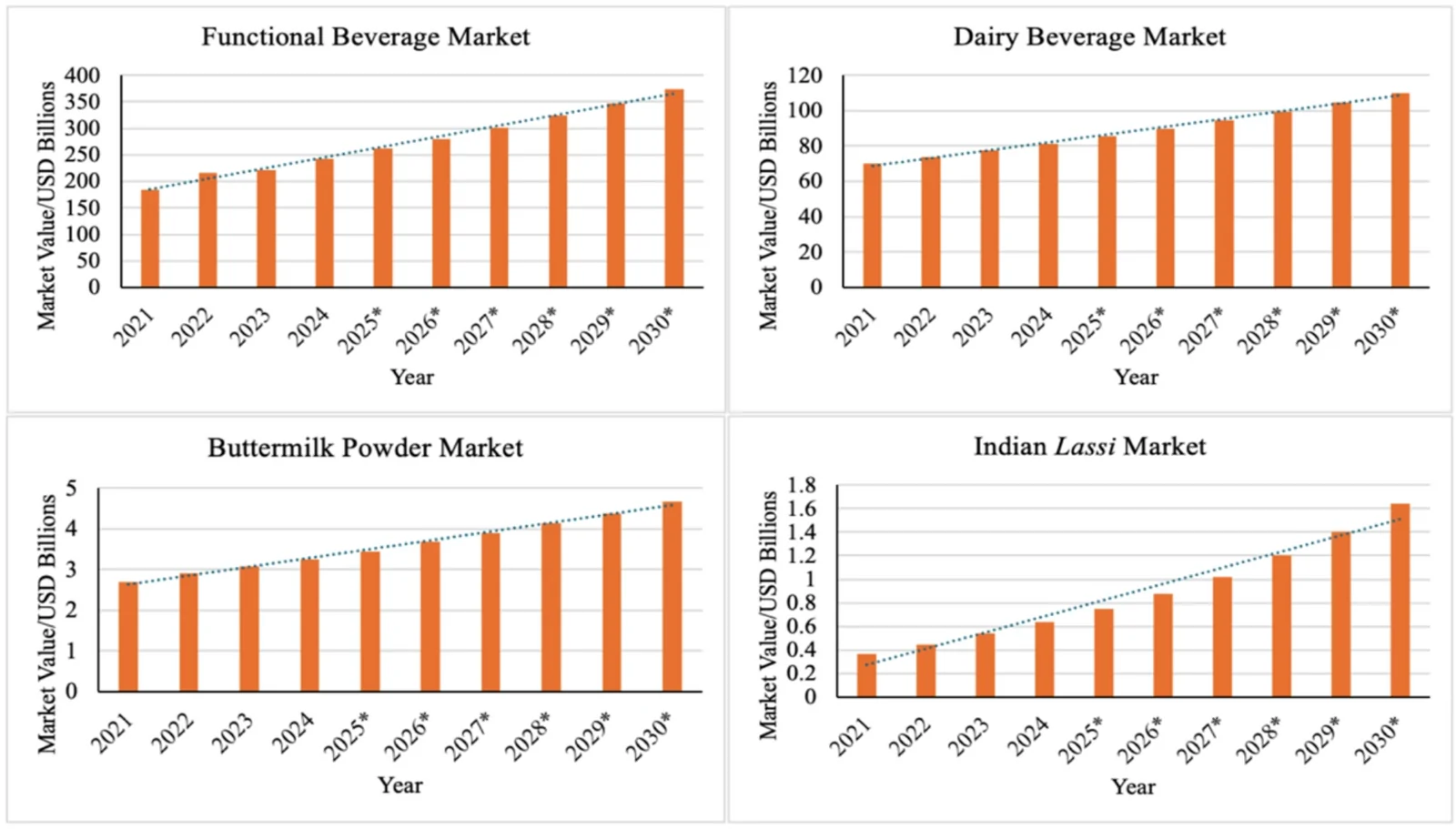
Graphical summary of the above-mentioned current market trends for various markets and their corresponding expected growth and market value between 2025–2030 as per cited sources. This aims to show the increase in demand for dairy-based and functional beverages globally, with Indian *Lassi *being a great example

Figure 9 summarizes projected 2021–2030 growth for functional beverages, dairy beverages, buttermilk powder, and the Indian *Lassi *market [87, 90, 94–99]. All sectors show upward trends, reflecting rising global demand for dairy-based and functional drinks. Projections for 2025–2030 were calculated from 2024 market values and CAGR estimates, highlighting strong growth potential for health-focused and traditional dairy beverages.

### Consumer Preferences

Consumer preferences for *Lassi *are shaped by cultural, sensory, and environmental factors, which have significant implications for product development and market trends. The choice of flavor profiles and packaging solutions reflects both regional traditions and global market demands.

Global *Lassi *preferences vary by region. Sweet *Lassi *dominates international markets, especially in North America and Europe, where flavors like mango, rose, and cardamom, led by mango, appeal to consumers of fruit-based, dessert-like drinks [100]. In South Asia, salty *Lassi *is preferred for its digestive and cooling properties, traditionally spiced with cumin, black salt, and green chili [1]. While its global share is smaller, it attracts niche health-focused markets. Emerging trends include hybrid flavors, such as turmeric-infused sweet *Lassi *and mint-spiced salty *Lassi *[101], as well as low-sugar and sugar-free sweet variants marketed for reduced calories and functional benefits [6].

Probiotic-enriched *Lassi*, particularly those containing strains like *Lactobacillus acidophilus* and *Bifidobacterium bifidum*, is increasingly preferred by health-conscious consumers. These products are advertised for their potential health benefits, including improved gut health, enhanced immunity, and reduced gastrointestinal inflammation. Moreover, advancements in probiotic stabilization and encapsulation techniques have enabled manufacturers to offer shelf-stable probiotic *Lassi*, addressing logistical challenges in distribution. This innovation enhances the accessibility of probiotic *Lassi *in both urban and rural markets, contributing to its rising popularity.

Packaging strongly influences *Lassi *purchase and consumption patterns. Single-serve formats like tetra packs and PET bottles dominate urban markets for their convenience, with the global single-serve packaging sector valued at USD 181.62 billion in 2022 and projected to reach USD 250.0 billion by 2032 [102]. Sustainability is driving innovation, with biodegradable and recyclable options favored in eco-conscious regions such as Europe and North America. Consumers view sustainable packaging positively, while resealable formats are gaining popularity for multi-use convenience and freshness [103].

In conclusion, consumer preferences for *Lassi *are evolving towards sweeter flavor profiles, innovative packaging solutions, and environmentally sustainable practices. These trends reflect broader shifts in global consumer behavior and are likely to influence future developments in the *Lassi *market.

### Rising Demand for Indian *Lassi*

Rising demand for fortified Lassi reflects consumer interest in beverages offering health benefits beyond basic nutrition. These products are enriched with vitamins (D, B12, A) and minerals (calcium, zinc, iron) [104, 105], supporting public health goals to combat micronutrient deficiencies, especially in developing nations.

Plant-based and vegan *Lassi *alternatives are growing in popularity, driven by rising lactose intolerance and vegan/flexitarian diets. Made with soy, almond, oat, or coconut milk, they mimic traditional *Lassi *while meeting dietary and ethical preferences [106]. These versions are often fortified with vitamin B12 and calcium to address nutritional gaps [107], and some use plant-derived probiotics to replicate functional benefits. The global plant-based beverage market is projected to grow at a 12.9% CAGR from 2021 to 2026, with North America leading at 12.7% [108]. This trend reflects the fusion of scientific innovation and evolving consumer demand, positioning *Lassi *as a versatile player in the functional beverage market.

### Export and Global Markets for Indian *Lassi*

The export of *Lassi *has seen significant growth as the global food and beverage market increasingly embraces ethnic, probiotic-rich, and functional beverages. *Lassi*, traditionally consumed in South Asia, has garnered attention worldwide, particularly as interest in health-conscious products continues to rise.

*Lassi *is exported to 104 countries with large South Asian diaspora communities, including the U.S., U.K., Canada, and Australia [89]. The U.S. yogurt and probiotic drink market was valued at USD 8.43 billion in 2022 and is projected to grow at an 8.7% CAGR through 2030 [97]. In the U.K., the ethnic food market is expected to expand from USD 9.47 billion in 2023 to USD 17.70 billion by 2032 (CAGR 7.10%) [109]. Canada and Australia remain strong export markets, while health-conscious demand in the EU is driving growth in probiotic beverages, projected to reach USD 45.61 billion by 2030 (CAGR 6.78%) [110]. Germany, France, and the Netherlands lead this segment [111]. Rising awareness of probiotics’ gut health benefits continues to boost *Lassi 's *global appeal.

Global demand for *Lassi *offers growth opportunities but also challenges in balancing authenticity with diverse tastes. Traditionally made from churned yogurt with specific cultures for tang and probiotics, *Lassi ’s* global versions often use plant-based alternatives like almond or coconut milk to meet vegan and lactose-free demand, altering texture and flavor [112]. Flavor adaptation is another hurdle. Salty *Lassi*, common in South Asia, is less appealing in markets like the U.S. and EU, where sweeter dairy drinks dominate. Most U.S. consumers prefer sweet, organically sweetened yogurt beverages over savory ones [113]. This has led producers to increase sugar or add fruit flavors such as strawberry, mango, and peach boosting market appeal but risking cultural authenticity.

Packaging poses another challenge for *Lassi ’s* global market. Traditionally served in earthenware or metal cups with cultural significance [114], exports now use plastic bottles, tetra packs, or glass jars for convenience, shelf stability, and cost efficiency [115]. While practical, these formats lose the cultural connection of traditional serving methods. Mintel’s 2020 global packaging trends [116] report stresses the value of designs that reflect cultural and environmental values, highlighting the need for packaging that balances tradition with modern market demands.

Regulatory requirements also affect *Lassi *exports. The U.S. FDA mandates specific labeling for probiotic-containing products, while the EFSA enforces strict limits on probiotic health claims [117]. Compliance demands extra resources and can restrict how *Lassi *is marketed internationally, posing challenges to maintaining authenticity.

## Challenges in Production and Commercialization of Indian *Lassi*

### Microbial Stability and Quality of Indian *Lassi*

Producing probiotic *Lassi *poses challenges in maintaining microbial stability and viability. Strains such as *Lactobacillus* and *Bifidobacterium* are sensitive to temperature, pH changes, oxygen, and competing microbes, leading to viability loss during storage, transport, and shelf life [118]. This is critical, as probiotic efficacy depends on delivering sufficient live microorganisms at consumption.

Microbial viability in probiotic *Lassi *depends on controlled conditions, often requiring lower temperatures than standard dairy products. High-temperature processes like pasteurization can sharply reduce probiotic counts. Proper strain selection, formulation, and processing can deliver high-quality probiotics in various formats [119]. Production also risks cross-contamination with pathogens like *E. coli* and *Salmonella*, which threaten safety and disrupt probiotic balance [120]. Preventing this requires strict hygiene, quality starter cultures, and sanitation protocols. Contamination can impair probiotic metabolism, causing quality loss, off-flavors, and reduced health benefits [121].

Probiotic stability in *Lassi *requires optimal carrier matrices and suitable packaging. Encapsulation can protect cells from oxygen, moisture, and light [122], while modified atmosphere packaging (MAP) or vacuum sealing reduces oxidative stress and extends viability [123]. Formulations should also include prebiotics and nutrients to sustain probiotic activity. A major challenge is balancing stability with palatability, as some strains cause undesirable flavors or lose viability in acidic environments [124]. Strains must be chosen for robustness and compatibility with *Lassi ’s* taste and texture.

### Regulatory Challenges of Indian *Lassi*

The production of probiotic beverages, including *Lassi*, is subject to a complex landscape of regulatory and legal requirements that vary across regions. Ensuring compliance with food safety standards, labeling regulations, and health claims is critical to maintaining consumer trust and product credibility while avoiding legal repercussions.

The commercialization of probiotic beverages such as *Lassi *requires adherence to a complex set of regulatory frameworks to ensure safety, quality, and accurate product representation. Regulatory bodies such as the Food Safety and Standards Authority of India (FSSAI) and the U.S. Food and Drug Administration (FDA) impose stringent standards to protect consumer health and foster market transparency. However, the regulatory approaches of these agencies differ significantly, posing challenges for manufacturers seeking to market *Lassi *and other probiotic beverages across international boundaries.

In India, the FSSAI regulates probiotic beverages under the 2023 Food Safety and Standards regulations, setting microbiological limits for pathogens like *E. coli*, *Salmonella*, and *Listeria*, and requiring ≥ 10⁸ CFU/g of viable cultures for functional status [125]. In the U.S., the FDA oversees probiotic beverages via the FDCA and FSMA, classifying them as functional foods or dietary supplements based on claims [117, 126]. HACCP protocols are mandatory, with a functional threshold of 10⁷ CFU/g at production, expected to retain 10⁶ CFU/g by end of shelf life. Scientific evidence of strain viability is encouraged. However, *Lassi *often averages around 10⁵ CFU/g, falling short of probiotic labeling requirements [127].

Labeling rules for probiotic beverages differ between India and the U.S. In India, FSSAI allows the term “probiotic” only if microbial content and strain ID meet its standards, requiring the strain name, CFU count at end of shelf life, and storage instructions on labels; health claims must be scientifically supported but face less stringent approval than in the EU or U.S [128]. In the U.S., the FDA regulates claims under the NLEA, permitting truthful structure-function claims like “supports digestive health,” while disease-related claims require pre-approval and strong clinical evidence. Labels must list CFU counts, storage conditions, and serving sizes [117, 126]. Noncompliance can result in recalls, fines, or legal action.

Differences between FSSAI and FDA regulations complicate marketing *Lassi *in both countries. In India, rules focus on microbial viability and compliance with fermented milk product standards [125], but enforcement is often inconsistent, especially among small and medium enterprises. In the U.S., regulations emphasize transparency and consumer protection, with stricter oversight of health claims and labeling. The lack of a unified FDA definition of probiotics creates ambiguity, and health claims require strong clinical validation, demanding significant R&D investment. Regulatory differences between India and the U.S. highlight the need for harmonized probiotic beverage standards. Global criteria for probiotic content, viability, and health claims could ease trade and boost consumer trust.

To overcome these regulatory challenges, partnerships with academic institutions and research organizations can help generate the scientific evidence required to substantiate health claims and support the introduction of a market-credible *Lassi *product in the USA.

## Prospects for Indian *Lassi*

### Innovations in Microbial Research

Advances in microbial research are enabling the development of Indian *Lassi *with enhanced health benefits and production efficiency. Novel next-generation probiotics (NGPs), such as *Eubacterium hallii*, *Faecalibacterium prausnitzii*, *Roseburia* spp., *Akkermansia muciniphila*, and *Bacteroides fragilis*, show potential for immune modulation, gut health, and metabolic benefits [129, 130]. Identified through high-throughput sequencing and functional genomics, these strains have been found in fermented foods like kimchi, beer, and kefir [131]. NGPs often tolerate heat, acidity, and oxygen better than conventional probiotics, making them a promising candidate for functional beverages like *Lassi *.

NGPs show promise for managing conditions like IBS, metabolic disorders, and mental health via the gut-brain axis. However, research on optimal strain selection, dosage, and interactions with *Lassi ’s* dairy or plant-based bases is still limited. Their use could enable personalized nutrition, tailoring probiotic strains to improve digestion, immunity, or mental wellness. A deeper understanding of NGP function in fermented matrices is essential for developing targeted, effective functional *Lassi *products.

Advances in genome sequencing and AI/ML predictive modelling enable precise optimization of *Lassi *fermentation by revealing genetic traits that influence flavor, texture, and probiotic activity [132, 133]. Studies have identified strains like *Lactococcus lactis* and *Lactiplantibacillus plantarum* with bile salt resistance and intestinal adhesion [134]. Genome editing tools such as CRISPR-Cas are being explored to enhance robustness, stress tolerance, and bioactive peptide production in *Lactobacillus* species [135], paving the way for designer probiotics and consistent, high-quality functional *Lassi *.

### Innovations in Consumer Preferences

The innovation in *Lassi *production has expanded its scope beyond a traditional fermented dairy drink into a versatile vehicle for functional and hybrid beverages. These advancements target the growing market for personalized nutrition and functional foods, leveraging both modern science and traditional practices.

Hybrid *Lassi *with fruit extracts like mango, pomegranate, strawberry, and blueberries combines enhanced flavor with bioactive compounds such as polyphenols, vitamins, and antioxidants [136]. Mango, rich in carotenoids and vitamin C, may complement probiotic benefits [137]. Incorporating Ayurvedic herbs like turmeric, Ashwagandha, Tulsi, and Ginger into *Lassi *enhances its therapeutic potential with anti-inflammatory, adaptogenic, antimicrobial, and digestive benefits [138]. Challenges include low solubility and stability of phytochemicals, which can be addressed through microencapsulation or emulsification [139]. Sensory trials are key to balancing herbal flavors with *Lassi ’s* traditional taste for consumer acceptance.

Hybrid and medicinal *Lassi *can appeal to consumers seeking targeted health benefits and natural wellness solutions. Research should optimize the synergy between probiotics, fruit extracts, and herbs, using tools like metabolomics and microbiome analysis for scientific validation. These innovations enhance *Lassi ’s* value and position it as a competitive global functional beverage, promoting both health and product diversification.

### Shelf-Life Extension of Indian *Lassi *with Prebiotics

*Lassi *spoilage is mainly driven by microbial growth, including yeasts, molds, and bacteria, which degrade taste, odor, and quality [67]. Post-acidification from lactic acid bacteria lowers pH, increases sourness, and destabilizes proteins [140]. Enzymatic activity from spoilage microbes can also degrade fats and proteins, producing off flavors. Maintaining probiotic viability, such as *Lactobacillus* and *Streptococcus*, is crucial for health benefits. The incorporation of prebiotics (e.g., inulin, oligofructose, galactooligosaccharides (GOS), lactulose, resistant starch) can extend *Lassi ‘s* shelf life through multifaceted mechanisms [141, 142] (Table 2; [142–150]).

**Table 2:** Potential prebiotics, with suggested concentration ranges and key considerations for inclusion in *Lassi*, as well as their predicted benefits. The prebiotics and their concentration range in *Lassi *are proposed based on studies of yogurt and other fermented dairy products

Prebiotic	Concentration	Potential Benefits	Potential Considerations
Inulin	~ 0.2–3%	Increased probiotic viability, improved texture (viscosity, creaminess), reduced syneresis.	Potential for off flavors at higher concentrations, impact on sweetness, may alter traditional consistency.
Oligofructose	~ 1–2%	Enhanced probiotic growth, improved mouthfeel, potential for increased sweetness.	May have less impact on viscosity compared to inulin, potential for digestive discomfort in sensitive individuals at higher doses.
Galactooligos- accharides	~ 0.3–6%	Promotes growth of *Bifidobacteria* and *Lactobacilli*, may improve texture and mouthfeel.	May not be suitable for individuals with milk allergies depending on the source.
Lactulose	~ 1–2%	Enhances probiotic survival, prebiotic effects.	Can have laxative effects at higher doses.
Resistant Starch	~ 2–4%	Improves texture, acts as a stabilizer and fat substitute, prebiotic benefits.	May affect the consistency depending on the type and concentration.

Firstly, prebiotics reinforce probiotic metabolic activity, increasing the production of lactic acid and antimicrobial compounds like bacteriocins and hydrogen peroxide, thereby inhibiting spoilage organisms [151]. Secondly, prebiotics foster a favorable microbial balance by promoting beneficial bacterial dominance, suppressing spoilage microorganism proliferation. Certain prebiotics, such as inulin and lactulose, can also modulate pH by accelerating acidification or maintaining controlled acidity, which, in moderation, hinders specific spoilage bacteria [147, 152]. Lastly, prebiotics enhance probiotic survival during storage by supplying an accessible energy source and potentially offering protective effects within the *Lassi *matrix [142].

Research on yogurt and other fermented dairy products supports prebiotic integration into *Lassi*. Inulin (0.2-3%) enhances probiotic viability and texture [143], with *Bifidobacterium bifidum* viability improving at 0.2–0.6% [143, 152] and sensory preference at 1% in *Lassi *[149]. While 2.5% inulin was acceptable in *misti dahi* (sweet yogurt), > 10% reduced quality [153]. Oligofructose (1–2%) also benefits bacterial populations and combining 2% inulin with 2% fructooligosaccharides (FOS) in yogurt extended shelf life and boosted probiotic counts [143].

In dairy applications, galactooligosaccharides (GOS) have been shown to improve probiotic survival in yogurt [144, 151]. Lactulose similarly enhances probiotic viability in yogurt at 1–2% concentrations, while higher dosages are employed for its general prebiotic properties [146, 148]. Resistant starch, when incorporated into dairy products at 2–4%, concurrently improves textural attributes and probiotic survival [145, 148].

Beyond their primary prebiotic functions, inulin, oligofructose, GOS, lactulose, and resistant starch also influence the physicochemical properties of *Lassi *and other fermented dairy products [142, 145, 148, 150]. Long-chain inulin, owing to its water-binding and gel-forming capabilities, notably increases viscosity and imparts a creamy texture, while shorter chain oligofructose has a less pronounced impact on viscosity but still contributes to improved mouthfeel. GOS similarly enhances texture and mouthfeel in dairy products. Furthermore, inulin’s ability to enhance water retention can reduce syneresis during storage. Resistant starch also plays a role, acting as a stabilizer and fat substitute, thereby influencing overall texture.

The incorporation of prebiotics will also influence the sensory profile of *Lassi *[142, 145, 148, 150]. Regarding sweetness, inulin imparts a mild sweetness (approximately 10% of sucrose), whereas oligofructose is considerably sweeter (30% to 60% of sucrose). GOS also contributes to sweetness. These inherent sweetening properties necessitate careful formulation of *Lassi *to avoid excessive sweetness. Both inulin and oligofructose further contribute to a smoother and creamier mouthfeel, enhancing the overall sensory experience. However, off flavors may occur at higher concentrations of inulin or in specific matrices. Lactulose possesses a sweetness profile comparable to sucrose, while resistant starch is generally neutral flavor.

The exploration of long-term health impacts and the incorporation of next-generation probiotics into *Lassi *are well linked. As new probiotic strains are introduced, their long-term effects on the human body, particularly when consumed daily, need to be studied to assess their sustainability and safety. Therefore, a scientific understanding of the synergistic effects of long-term consumption of NGPs in *Lassi *could lead to the development of probiotic products that offer not only immediate benefits, such as gut health and immune modulation, but also sustained positive impacts on metabolic health, cognitive function, and overall well-being. Further research in this area could help identify the most suitable NGP strains, dosages, and consumption patterns that optimize health benefits while ensuring that these products remain safe and beneficial over extended periods of use.

## Conclusion

Indian *Lassi *remains a significant part of traditional South Asian diets while evolving into a globally recognized functional beverage. Its probiotic content and rich nutritional profile contribute to its health benefits, including improved digestion, gut health, and immune support. *Lassi ’s* cultural relevance extends beyond consumption, as it plays a role in religious practices, seasonal traditions, and regional culinary diversity. However, challenges remain, including addressing regulatory barriers and ensuring microbial viability throughout storage and distribution. On the contrary, Indian *Lassi *is known to host a very limited number of probiotic strains, approximately between 5 and 8 strains. Additionally, due to lower acidity of *Lassi *as compared to Kefir and Kombucha, the shelf-life also reduces. This has primarily affected the marketing of *Lassi *as a functional probiotic drink.

Unfortunately, based on our current knowledge, there is no previous research that has been conducted to improve the probiotic diversity of Indian *Lassi *to better understand its long-term health benefits and its potential to be advertised as a functional probiotic beverage in the Western world. Future research should focus on optimizing probiotic formulations, improving sustainable production techniques, and investigating the long-term health effects of *Lassi *consumption. By bridging traditional knowledge with modern scientific advancements, *Lassi *has the potential to become a staple in the global functional food industry, benefiting both health-conscious consumers and the food industry. 

## Data Availability

Data will be made available on request.
